# Phloem wedges in Malpighiaceae: origin, structure, diversification, and systematic relevance

**DOI:** 10.1186/s13227-022-00196-3

**Published:** 2022-04-28

**Authors:** Angélica Quintanar-Castillo, Marcelo R. Pace

**Affiliations:** 1 Present Address: Posgrado en Ciencias Biológicas, Instituto de Biología, Universidad Nacional Autónoma de México, Circuito Zona Deportiva s.n. de Ciudad Universitaria, Coyoacán, 04510 Mexico City, Mexico; 2 Present Address: Departamento de Botánica, Instituto de Biología, Universidad Nacional Autónoma de México, Circuito Zona Deportiva s.n. de Ciudad Universitaria, Coyoacán, 04510 Mexico City, Mexico

**Keywords:** Cambial variant, Discontinuous cambium, Lianas, Limiting rays, Malpighiaceae, Phloem, Phloem wedges, Vascular cambium, Xylem

## Abstract

**Background:**

Phloem wedges furrowing the wood are one of the most notorious, widespread types of cambial variants in Angiosperms. Many lianas in Malpighiaceae show these variations in the arrangement of the secondary tissues. Here we explore their ontogeny, structure, and evolution in Malpighiaceae, where phloem wedges appeared multiple times, showing how they have contributed to the anatomical diversification of the family. Using a broad sampling with 143 species from 50 genera, covering all major lineages in Malpighiaceae, we crossed data from ontogeny, stem anatomy, and phylogenetic comparative methods to determine ontogenetic trajectories, final anatomical architectures, and evolution within the most recent phylogeny for the family.

**Results:**

Phloem wedges appeared exclusively in lianas and disappeared in shrub lineages nested within liana lineages. At the onset of development, the vascular cambium is regular, producing secondary tissues homogeneously across its girth, but soon, portions of the cambium in between the leaf insertions switch their activity producing less wood and more phloem, initially generating phloem arcs, which progress into phloem wedges. In the formation of these wedges, two ontogenetic trajectories were found, one that maintains the continuity of the cambium, and another where the cambium gets dissected. Phloem wedges frequently remain as the main cambial variant in several lineages, while in others there are additional steps toward more complex cambial variants, such as fissured stems, or included phloem wedges, the latter a novel type of interxylary phloem first described for the family.

**Conclusions:**

Phloem wedges evolved exclusively in lianas, with two different ontogenies explaining the 10 independent origins of phloem wedges in Malpighiaceae. The presence of phloem wedges has favored the evolution of even more complex cambial variants such as fissured stems and interxylary phloem.

**Supplementary Information:**

The online version contains supplementary material available at 10.1186/s13227-022-00196-3.

## Background

Compiling and deciphering the processes that generate morpho-anatomical diversity is at the core of evolutionary biology. Here, we combine ontogeny and phylogeny to unravel how, when and under which circumstances phloem wedges have appeared in Malpighiaceae, a pantropical family and one of the top 10 most abundant lianescent families of the Neotropics [1, 2]. Malpighiaceae comprise 1300 species distributed in 77 genera [3], with South America being their center of diversity, and paleotropical members deriving from at least seven independent long-distance dispersion events from the Neotropics [4–6]. The lianescent habit is a feature that was suggested to have evolved multiple times within the family, entailing considerable changes in stem development both in their external morphology, by the formation of twining stems, and in their vascular system conformations [7–9].

In Malpighiaceae, many lianas have a regular secondary growth, but an equally large number display considerable changes in their vascular configurations that produce different arrangements and amounts of secondary xylem and phloem in their stem, resulting from cambial variants [10–13]. Cambial variants are alternative forms of secondary growth that are especially common in lianas and have been shown to increase the flexibility for climbing and act in wound repair and regeneration, given the common presence of non-lignified parenchyma prone to division and re-differentiation in these variants [7, 14, 15].

Interest in cambial variants in Malpighiaceae has been documented since the nineteenth century [16–26]. Due to this interest, five different types of cambial variants have been recognized in Malpighiaceae: (a) interxylary phloem in *Dicella*; (b) non-cylindrical/lobed stems described for some species of *Heteropterys*; (c) phloem wedges, such as in *Mascagnia,* and *Peixotoa*; (d) fissured stems, such as in *Alicia* and *Flabellaria* and (e) interxylary cambia, described for *Stigmaphyllon* and *Banisteriopsis* serie *nummifera* [23, 27, 28]. However, the most widespread cambial variant in Malpighiaceae is by far the xylem furrowed by phloem wedges [21, 25] a variant that has been also named “interrupted xylem” by other authors [29, 30].

Phloem wedges typically derive from a switch in the activity of certain discrete portions of the vascular cambium. These now variant portions start to produce less secondary xylem and more secondary phloem. The differences in the production of secondary tissues initially form phloem arcs, which eventually become deep phloem wedges [10, 31]. These differences in cambium production are related to a change in the products of the cambium and alterations in the rates of periclinal and anticlinal divisions. While in the variant portions anticlinal divisions are in some cases reduced, periclinal divisions produce more phloem than xylem practically at the same rates that the regular portions continue producing more xylem than phloem. Hence, there is a discontinuity of products between these two co-occurring portions, but an almost perfect synchronization of the cambial divisions, forming a symmetrical stem, even if non-cylindrical. This discontinuity suggests that secondary growth is regulated by modules [32].

Despite their common occurrence in many angiosperm families such as Asteraceae, Bignoniaceae and Sapindaceae ([12, 13, 33], check [10] for a complete list), the developmental mechanisms and phylogenetic distribution of phloem wedges in Malpighiaceae have never been explored. Here we carried a broad sampling across the entire phylogeny of the Malpighiaceae and selected key species, for a thorough ontogenetic study, to explore where phloem wedges are present, how they are ontogenetically formed, and if their development pathway is common or different among the various lineages.

This is the first study of phloem wedges within a family where this feature seems to have evolved in distantly related genera, opening new avenues to understand possible triggers of this cambial variant, their differences, and commonalities in different genera, and what their impact may be in the process of diversification of a large plant family. The aims of this study, therefore, are (1) to explore if the evolution of phloem wedges occurred in a convergent, conserved, or random fashion within the family; (2) to determine if there is a relation between the habit and phloem wedges presence; (3) to describe the structure, ontogenetic processes, and developmental mechanisms involved in the formation of phloem wedges in different lineages of Malpighiaceae.

## Methods

### Taxon sampling

We collected and analyzed 145 species belonging to 52 genera of Malpighiaceae, representing all the major clades in the family according to the phylogeny of Anderson and Davis [3], except for *Ectopopterys,* a monotypic lineage of lianas from northern South America (Additional file 1: list S1). Of all the species collected, we selected 12 with xylem furrowed by phloem wedges to study ontogenetically. These species and their respective clades were *Alicia anisopetala*, *Christianella multiglandulosa*, *Mezia mariposa* (Christianella clade), *Carolus chasei* (Carolus clade), *Hiraea fagifolia* (Hiraea clade), *Mascagnia sepium*, (Malpighia clade), *Niedenzuella multiglandulosa* (Niedenzuella clade), *Diplopterys pubipetala*, *Janusia guaranitica, Peixotoa sericea* and *Stigmaphyllon acuminatum* (Stigmaphyllon clade), and *Tristellateia greveana* (Bunchosia clade).

Of these 52 genera, 42 occur in America (129 species) and 9 genera occur in Africa and Asia (15 species). Collections were mostly performed in natural populations from Mexico to Argentina and Madagascar, trying to cover the different biomes where Malpighiaceae is known to be present (e. g., Atlantic rainforest, Amazon, *Cerrado*, Chaco, deserts, and xeric shrublands). Vouchers for all specimens were deposited in one or more of the following herbaria CEPEC, COR, CTES, F, HUEFS, K, L, MEXU, MICH, MO, NY, P, RBR, SP, SPF, TAN, TUS, TWT, U, US and WAG, acronyms according to Thiers [34] (see Additional file 1: list S1 for species authorships, collector, collector number, localities, and where vouchers are deposited).

A few species, especially from Africa and Asia, were obtained through donations from the wood collection of Leiden (Naturalis, The Netherlands) and the wood collection of the Tsukuba Forestry and Forest Products Research Institute (TWTw), Japan.

### Anatomical analysis/procedures

For the ontogenetic analysis, we sampled, from the apex to the thickest part of the stem, node by node until the thickest part. Whenever possible, at least 3 individuals per species were collected. Samples were immediately fixed in FAA 50 [35] in the field and later transferred to 70% ethanol to preserve fragile tissues such as non-lignified parenchyma and phloem. In the case of material from wood collections, dried samples were rehydrated in boiling water and glycerin (1:10) [36, 37].

To corroborate the presence of phloem wedges and show them macroscopically, the stems were polished with sandpaper underwater before being photographed [38]. For the anatomical sections, because the woods of Malpighiaceae are particularly stiff, the stems were softened in a solution of boiling water and glycerin 1:1 for at least 1 week (8 h/day) [36] or hydrofluoric acid (HF) for 2 days [35].

After softening, we gradually embedded the samples in polyethylene glycol 1500 [39]. When completely embedded, the samples were placed in paper molds and then sectioned with the aid of a sliding microtome (Leica Hn40). We used permanent steel knives sharpened with sandpaper [40], a polystyrene resin coat (Styrofoam dissolved in butyl acetate), and adhesive tape to avoid tearing the tissues and to obtain entire sections [41]. Sections were double-stained in safranin and Astra-blue [42] and mounted either in a synthetic resin or Canada balsam to make permanent slides.

### Phylogenetic analysis

We produced a time-calibrated phylogeny to estimate the evolutionary history of habit and phloem wedges. We mainly used sequences derived from previous phylogenies for the family downloaded from GenBank [3, 6], adding some additional taxa not included in previous works, prioritizing the inclusion of species for which we had data on both their habits and stem anatomies. The data set consisted of three plastids (*mat*K*, ndh*F*, rbc*L) and one nuclear marker (*PHYC*). One hundred and fifty-four taxa represent the main clades in Malpighiaceae, plus 13 outgroup species belonging to Malpighiales and Saxifragales. The sampled species and GenBank accessions are listed in Additional file 1: List S2.

Sequences of each marker were aligned individually by manual refinements in Mesquite 3.6 [43]. We evaluated evolutionary models for each dataset separately and in combination using jModelTest 2 [44] and the Akaike information criterion (AIC). To check the concordance of the phylogeny with previously published ones, we analyzed maximum likelihood (ML) using RAxML-HPC v8 [45] as implemented in the CIPRES Science Gateway [46]. We implemented a GTR + I + Γ model as determined by the AIC, with 1000 bootstrap replicates, substitution parameters were estimated independently for the four data partitions and *Peridiscus lucidus* (Saxifragales) was specified as the outgroup.

To estimate the divergence times of Malpighiaceae, we conducted a Bayesian analysis with Beast 2.6.6 [47]. The calibrations used were the same as a previous time-calibrated phylogeny for Malpighiaceae [6] except for the root node. To calibrate the root node, we applied a secondary calibration derived from a detailed study of the rise of the main angiosperm families [48], corresponding to the crown age of Saxifragales + Vitales + Rosids. We applied a uniform prior distribution where the maximum value of the distribution was 125.3 Ma, and the minimum value was 119 Ma corresponding to the credibility interval (95% HPD). Dating analyses were conducted in BEAUTi with an uncorrelated-rates relaxed clock model obtained from a log-normal distribution (UCLN) [49]. The nucleotide substitution was under a GTR + I + Γ model, allowing independent estimation of parameters for each partition and the tree prior was under a birth–death model. We ran three independent Markov Chain Monte Carlo (MCMC), each with 350 million generations, sampling every 5,000 generations. The analyses were conducted in the CIPRES Science Gateway (45). We checked the convergence of the MCMC in Tracer 1.7.1 [50] and the parameter acceptance where the Effective Sample Size (ESS) was equal to or higher than 200 in most cases or > 100 for all the parameters. Files containing the sampled trees the MCMC runs were combined with LogCombiner. We obtained the Maximum Clade Credibility (MCC) tree with TreeAnnotator (Additional file 2).

### Phylogenetic comparative methods

We built a database with two discrete characters indicating the habit of the species (self-supporting/climber) and the presence of phloem wedges (presence/absence) in all studied species (check Additional file 3 for a description of characters). Character states were scored mainly from our own observations of adult specimens and preparations, summed to information from labels of digitalized herbarium vouchers nowadays widely available online, and from literature (floras or taxonomic treatments).

For each character, we estimated the character transition rate and determine the best-fit model (equal rates or all rates different) using a likelihood ratio test and the Akaike information criterion with fitMk function in the phytools R package [51, 52]. Using the best-fit model of evolution, we reconstructed the evolutionary history of both characters using the stochastic character mapping approach [53] on the MCC tree. We use the make.simmap function with 100 iterations, the results were summarized with the describe.simmap and countSimmap functions, and each character history was visualized in a single tree, adding the posterior probabilities at nodes using the plotSimmap function in phytools [51]. All model statistics are reported in the Additional file 4**.**

To test if phloem wedges evolved in correlation to the lianescent habit we performed a Pagel correlation test for discrete character evolution [54], using the fit.pagel function implemented in phytools [51]. Phylogenetic signals for each character were calculated on the MCC tree, using both Pagel’s λ and Blomberg’s K indices [55, 56] using the phylosig function in the phytools package [51] (Additional file 4).

### Terminology adopted

**Fig. 1:**
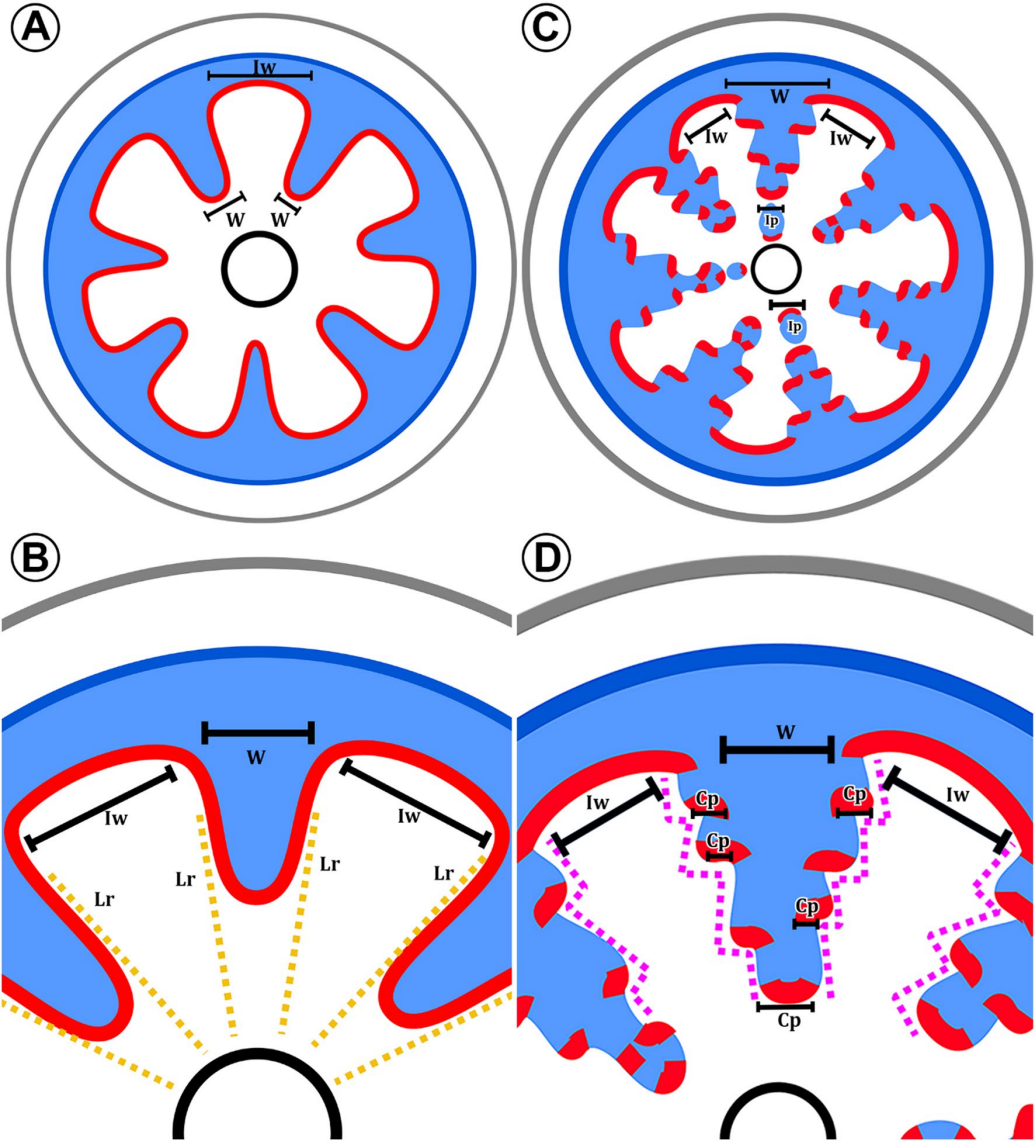
Terminology adopted for phloem wedges. **A** Stem with phloem wedges well-developed, two regions are identified: interwedges (Iw) and phloem wedges (W). Interwedges are the areas between phloem wedges and where the cambium maintains its regular activity. Phloem wedges are regions derived from a variant cambial portion that produces more secondary phloem and less secondary xylem. **B** Phloem wedge (W). Phloem wedges are commonly flanked by limiting rays (Lr), which are tall, wide multiseriate rays. **C** Phloem wedges well-developed with cambium disrupted. Mechanical pressure causes the inclusion of inner portions of wedges, known as interxylary phloem (Ip). **D** The transformation of flanking regions of the wedge from a regular to a variant activity causes wedges to have a stepwise pattern. Magenta dotted line outlines this pattern. Portions of the variant cambium (Cp) remain on the bottom of the wedges. Color key: black = pith limits, blue = secondary phloem, grey = dermal tissue, red = vascular cambium, orange = limiting rays

- *Variant cambium*. The cambium that changes its activity, and starts producing less xylem and more phloem, resulting in phloem wedges (Fig. 1A).
- *Regular cambium*. The cambium that maintains its original activity on the interwedge regions (Fig. 1A).
- *Interwedges*. The regions between phloem wedges and where the cambium maintains its regular activity, while phloem wedges are the variant areas (Fig. 1A).
- *Limiting rays*. A term coined by Schenck [21] to refer to broad, multiseriate rays on each side of the phloem wedges (Fig. 1B).
- *Interxylary phloem*. (Fig. 1C) Referred to portions of phloem embedded within the secondary xylem, which are produced by a single cambium [57]. Although it can have different ontogenetic origins, here we refer to the one where the phloem strands keep portions of an active cambium at its inner part.
- *Stepwise pattern*. A term that here describes the shape of the phloem wedges in *Tristellateia*, where portions next to the phloem wedges subsequently switch from a regular to a variant activity, forming steps (Fig. 1D).

## Results

### Phylogenetic distribution of phloem wedges in Malpighiaceae

**Fig. 2:**
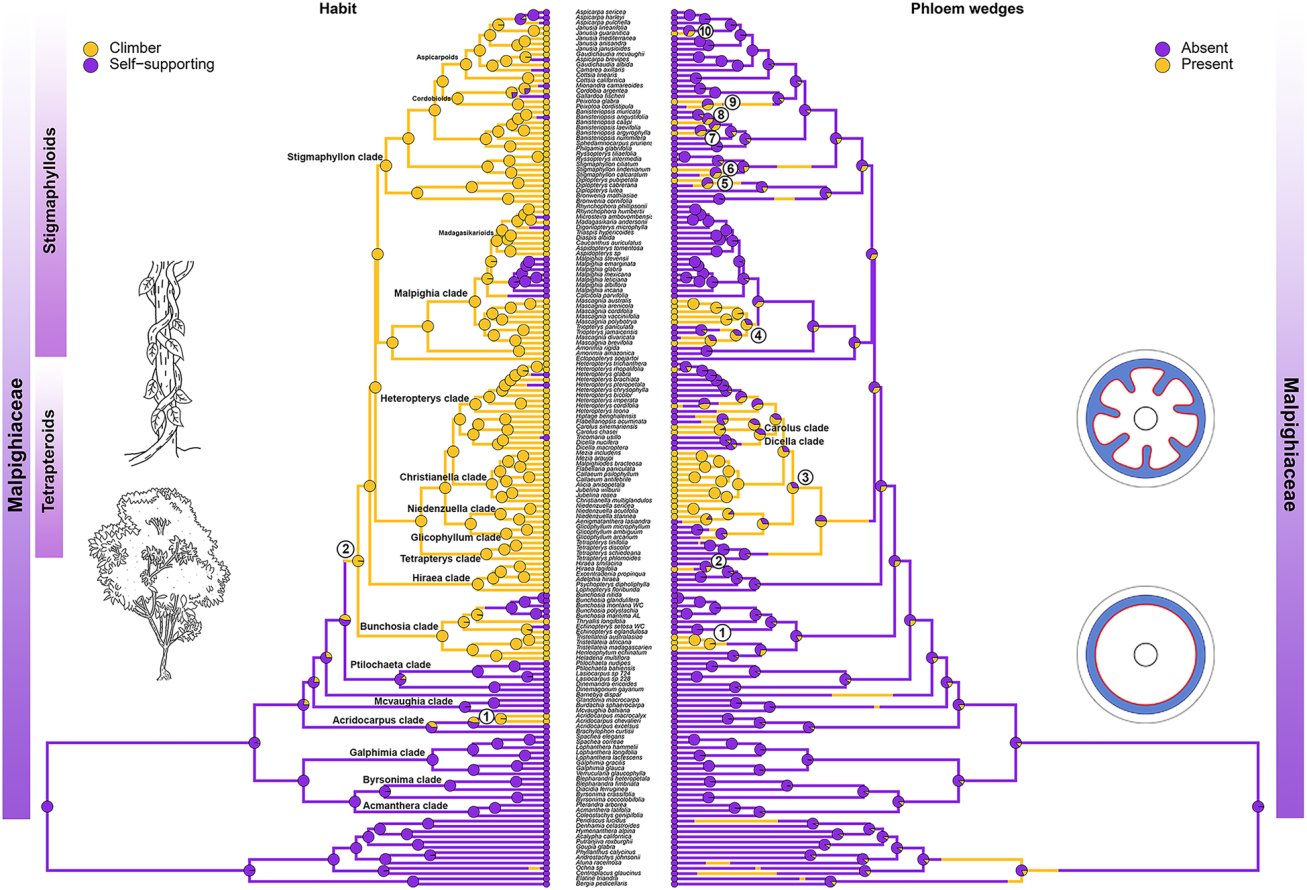
Ancestral character state reconstruction of lianescent habit and phloem wedges in Malpighiaceae. Trees show the occurrence of lianas (left) and stem with xylem furrowed by phloem wedges (right) in Malpighiaceae. Pie charts on nodes show the posterior probabilities of every state. For habit reconstruction (left), the purple color represents self-supporting plants (trees, shrubs, and sub-shrubs) and yellow represent climbing plants. For phloem wedges reconstruction (right) purple color represents the absence of phloem wedges and yellow its presence. Climbing plants appear twice in Malpighiaceae while phloem wedges appear ten times. Most of the phloem wedges are concentrated in tetrapteroids and stigmaphylloids, except for *Tristellateia* (Bunchosia clade) and *Hiraea* (Hiraea clade)

Phloem wedges have evolved exclusively in lianescent lineages, with at least 10 independent origins in Malpighiaceae within eight clades (Fig. 2). This variant evolved once in the Bunchosia clade (*Tristellateia*), once in the Hiraea clade (*Hiraea*), once in the ancestral node of Tetrapteroids (Niedenzuella clade (*Niedenzuella*), + Christianella clade, + Carolus clade (*Carolus*), + Heteropterys clade (*Heteropterys*)), once in the Malpighia clade (*Mascagnia*) and six times within the species-rich Stigmaphyllon clade (*Peixotoa*, *Diplopterys*, some *Banisteriopsis*, some *Stigmaphyllon*, and *Janusia*).

The self-supporting was inferred to be the ancestral condition for Malpighiaceae, while the evolution of lianescent habit likely occurred twice in the family. Lianas evolved once in the Acridocarpus clade and once in the ancestral node of Bunchosia clade + Tetrapteroids + Stigmaphylloids, being the most common habit in the family. Many reversions back to the self-supporting habit occurred, two of them being noteworthy for leading to large neotropical genera within Malpighiaceae, *Bunchosia,* and *Malpighia* (Fig. 2).

The ancestral state estimation indicates that phloem wedges are a derived character in the family being present only in lianas. The Pagel 1994 test showed support for the dependent model of correlated evolution (*p* = 0.0000076), indicating the evolution of phloem wedges was contingent on the evolution of lianas. Except for *Tristellateia* (Bunchosia clade) and *Hiraea* (Hiraea clade), all other genera are in two large clades known as tetrapteroids and stimaphylloids (Fig. 2). Some of these lineages have members that reversed to the self-supporting habit (e.g., *Heteropterys*, *Hiraea*, *Peixotoa*), and the cambial variants are absent on them, giving further support to the results of our correlation analysis.

For members of Christianella clade, the genus *Diplopterys,* and some species of *Banisteriopsis* and *Heteropterys*, phloem wedges represent one of the stages of their development, which later progresses into fissured stems. In *Tristellateia* and *Niedenzuella*, interxylary phloem is formed right after phloem wedges formation. Exploring the formation of fissure stems in detail is beyond our goal here because their formation has been surveyed in previous works, which will be treated in the discussion.

### Structure and development of phloem wedges

#### Phloem wedges external appearance and their position in the stems

**Fig. 3:**
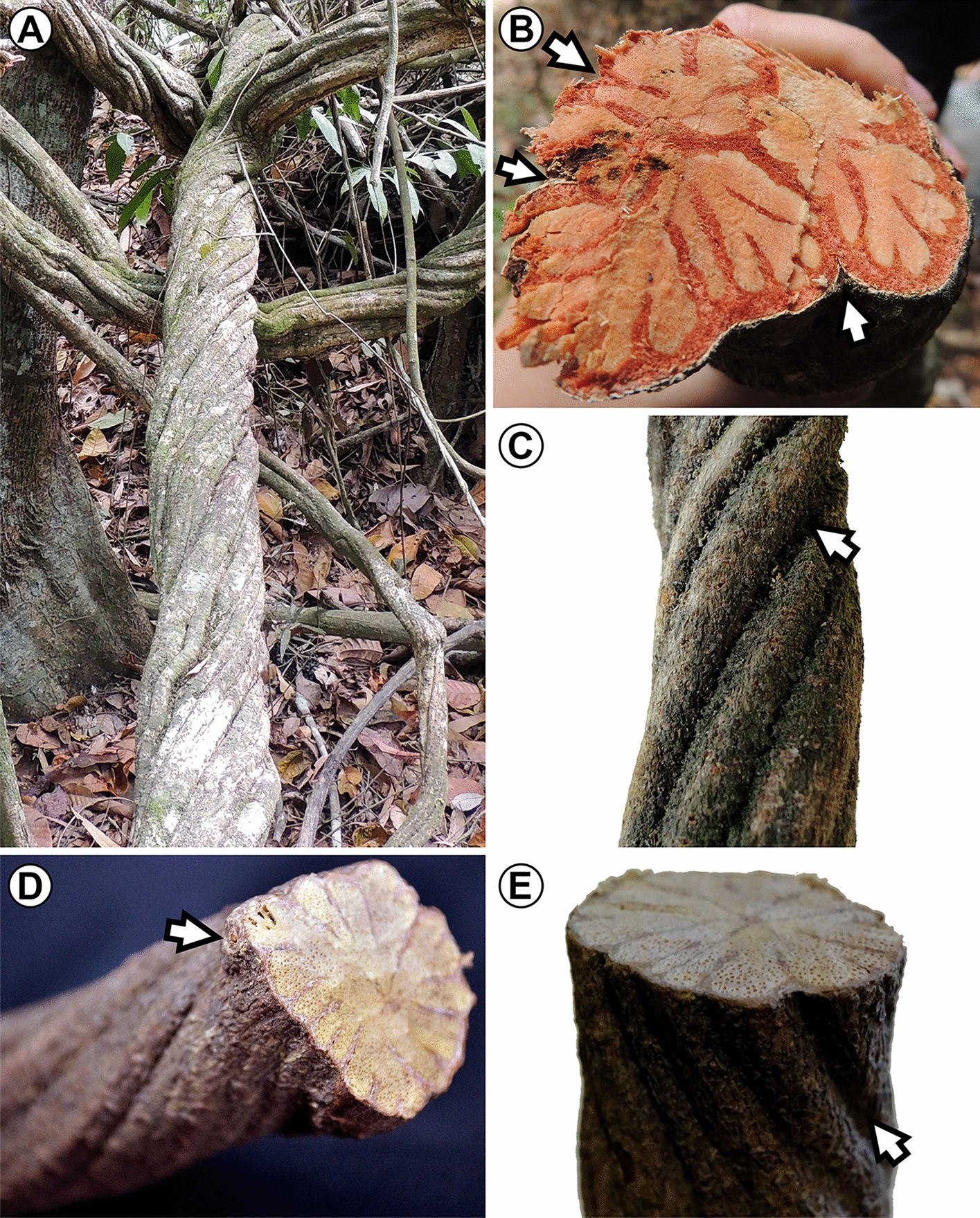
The external appearance of stems with xylem furrowed by phloem wedges. **A**, **B** *Heteropterys cordifolia*. **A** Thick stem of *H. cordifolia* holding onto adjacent trees and its own younger branches. **B** Cross-section of *H. cordifolia* stem showing the match of stem depressions with a phloem wedge (arrows). **C–E** *Mascagnia sepium*. **C** Phloem wedges can be detected morphologically since they generate concavities visible in the stem surface (arrow). **D** Phloem wedges match with the leaves in the stem (arrow showing leaf scar), and progress in a spiral fashion. **E** Coincidence of a stem depression with the development of a phloem wedge (arrow)

Macro-morphologically, the presence of phloem wedges can be perceived from the outside in most species (Fig. 3). In these cases, the stems exhibit depressions where the phloem wedges develop, as we here illustrate in *Heteropterys cordifolia* (Fig. 3A, B) and *Mascagnia sepium* (Fig. 3C–E), where the depressions are so marked that their stems become non-cylindrical. In some lianas, such as *Banisteriopsis caapi* (popularly known as one of the main ingredients of the ritualistic Ayahuasca), the phloem wedges can merge with dividing non-lignified parenchyma to join the pith and produce fissured stems, with an architecture similar to that seen in *Heteropterys cordifolia* (Fig. 3B). In contrast, in some specimens of *Tristellateia greveana*, the stem remains with a round outline and shows no depressions, despite having deep phloem wedges. It is noticeable that the initial phloem wedges are located in between leaf insertions, with no phloem wedges under the leaves (Fig. 3D).

#### Two main ontogenies of phloem wedges are present in Malpighiaceae

**Fig. 4:**
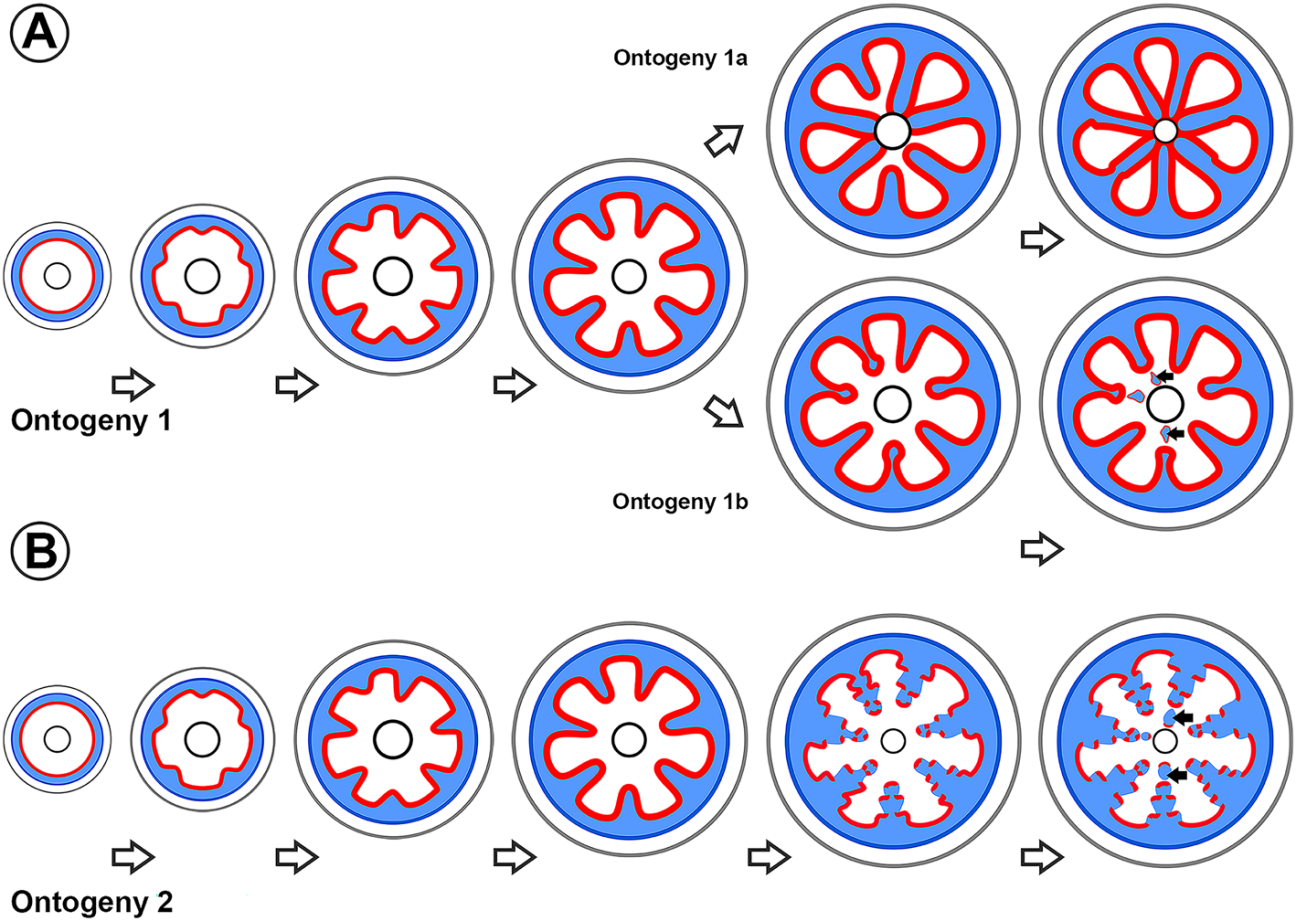
Two different ontogenetic trajectories are described in stems with xylem furrowed by phloem wedges in Malpighiaceae. **A** Ontogeny 1. Stems with phloem wedges that keep a single and continuous cambium (red line). After phloem wedges keep growing, these can be further developed into fissured stems (ontogeny 1a), or the tissue produced by regular cambia adjacent to the wedges may exert mechanical pressure including portions of the wedge (ontogeny 1b), this interxylary phloem keeps reminiscences of the active vascular cambium (black arrows). **B** Stems with phloem wedges that disrupt the cambium continuity and include portions of phloem wedges, resulting in interxylary phloem (black arrows). Color key: black = pith, blue = secondary phloem, grey = cortex, red = vascular cambium

Although we have detected 10 independent evolutions of phloem wedges in Malpighiaceae, we can group them under two main ontogenetic types (Fig. 4): (i) those which form phloem wedges with a single, continuous cambium (Fig. 4A); (ii) those whose variant cambia cease anticlinal divisions, disrupting the cambium continuity, leading to variant cambial portions, not in contact with the cambium of the regular portions (Fig. 4B). In the first type of ontogenetic trajectory, phloem wedges can be a stage in stem development that progresses to form fissured stems (Fig. 4A, ontogeny 1a) or included phloem (Fig. 4A, ontogeny 1b). In both ontogenies, the inclusion of portions of the phloem wedges into the xylem can occur, resulting in interxylary phloem (note the last stage in ontogeny depicted as Fig. 4A ontogeny 1b and Fig. 4B, ontogeny 2). Each of these two cases will be discussed in detail below.

#### Phloem wedges maintain a continuous cambium during their development (Ontogeny I)

**Fig. 5:**
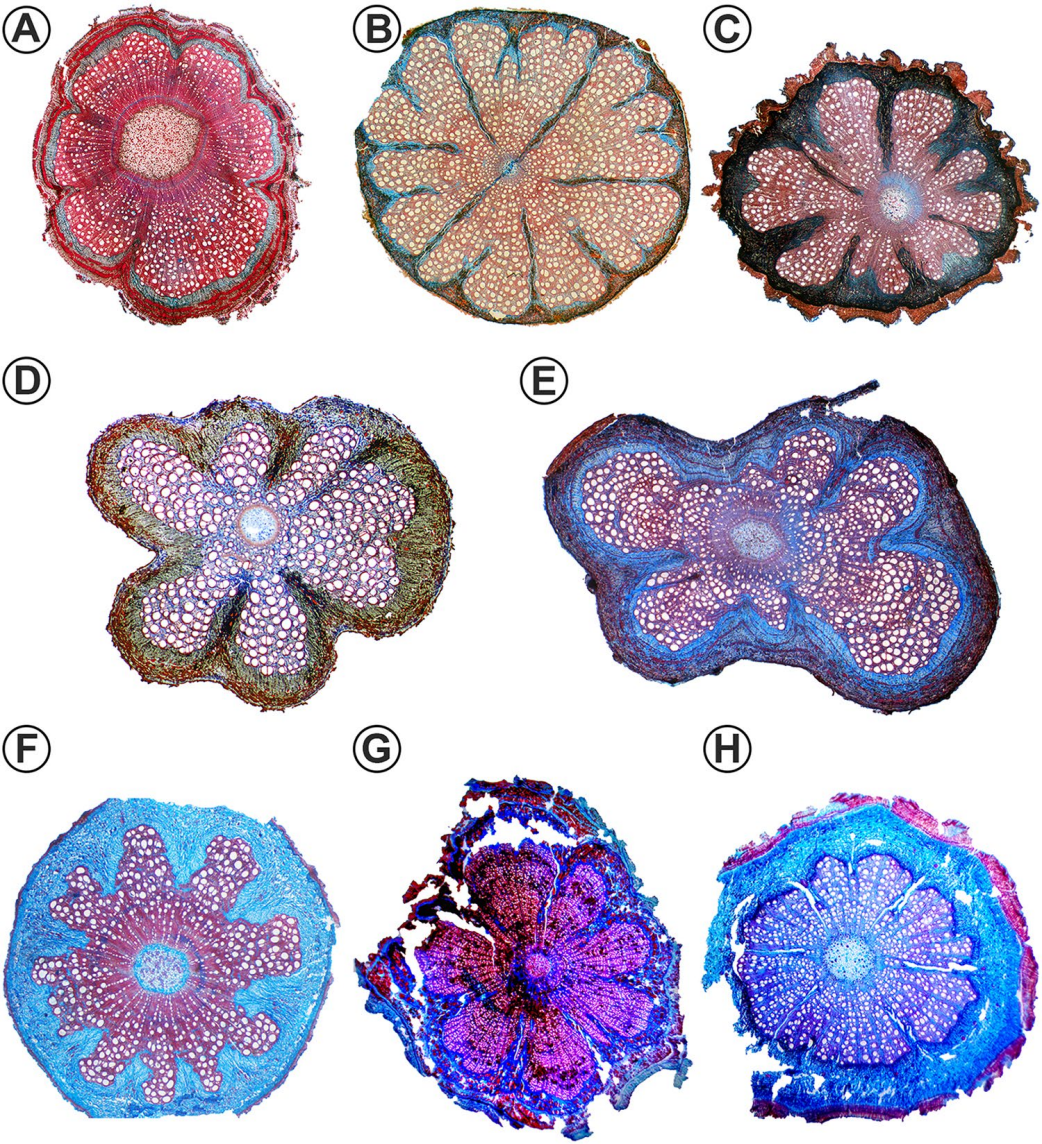
Light micrographs of stem cross-sections in Malpighiaceae showing phloem wedges diversity. All sections were double-stained in safranin and Astra-blue. **A** *Peixotoa sericea* with 6 phloem wedges. Note xylem under the phloem wedges is different from that of interwedges, with fewer vessels and more fibers. **B** *Mascagnia sepium* showing approximately 15 phloem wedges some deeper than the others, because of their different formation onset times. **C** *Niedenzuella multiglandulosa* showing 8–12 phloem wedges of different ages, some being deeper than the others **D** *Stigmaphyllon blanchetii* showing 6 phloem wedges generating marked concavities in the stem. **E** *Mascagnia divaricata* showing at least 4 well-formed phloem wedges. **F** *Alicia anisopetala* showing 9 well-formed phloem wedges. **G** *Tristellateia greveana* showing 8 phloem wedges, without apparently forming depressions in the stem. **H** *Tristellateia australasiae* showing 8 well-formed phloem wedges slightly marking concavities on the stem. Images not to scale

**Fig. 6:**
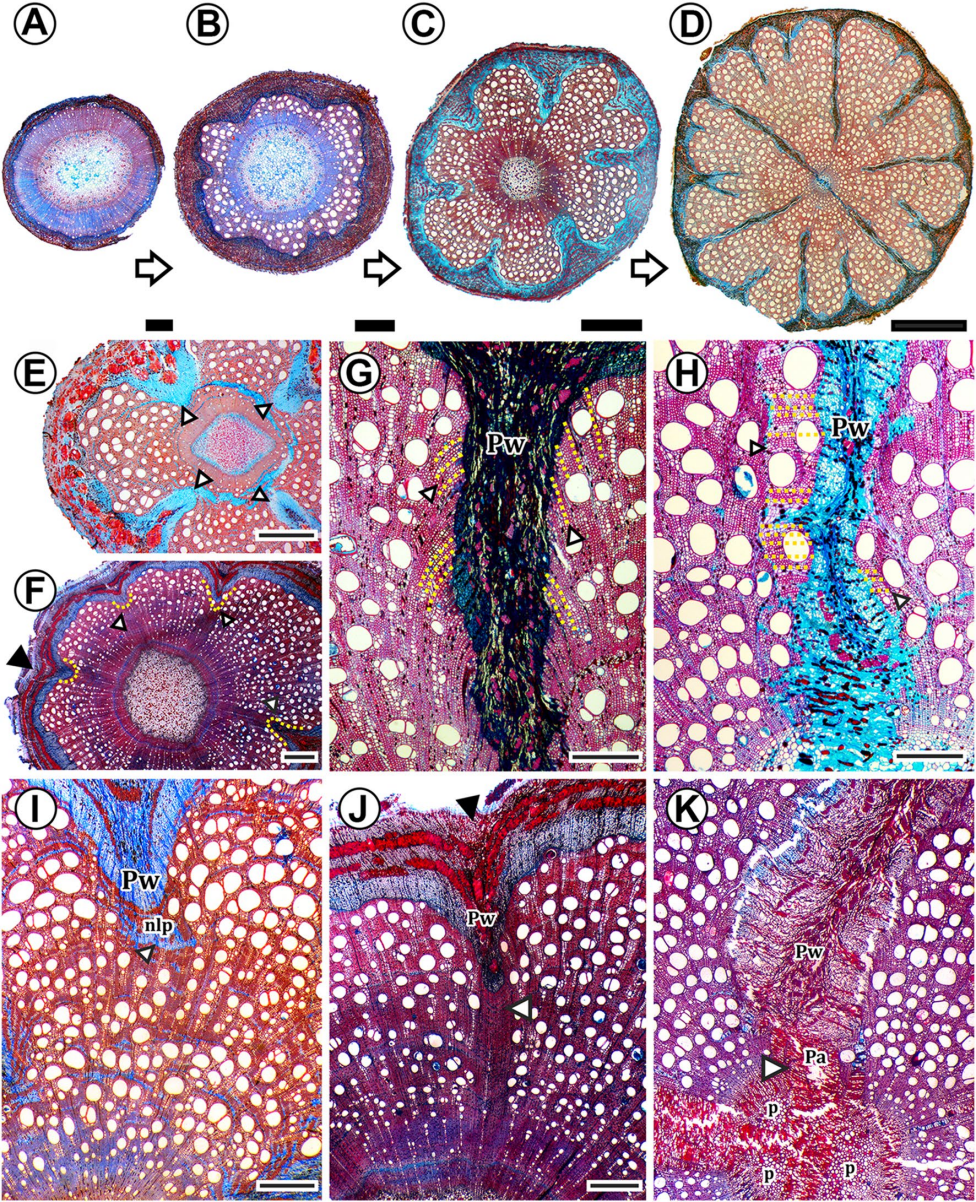
Light micrographs show the formation of phloem wedges that preserve a continuous cambium (Ontogeny I). All sections were double-stained in safranin and Astra-blue. **A–D** Development of the stem of *Mascagnia sepium.* **A** Onset of development with regular secondary growth. **B** Shallow arcs of phloem are formed approximately 6 to 8 shallow arcs are visible. **C** Phloem arcs get deeper and turn into phloem wedges, some deeper than the others, given their different time of onset formation. **D** Adult stem with deep phloem wedges that furrow the xylem, some deeper than the others. **E** *Mezia mariposa* phloem wedges (arrowheads). **F** *Peixotoa sericea* with phloem wedges with different stages of development, some deeper than others (yellow dotted line and arrowheads), phloem wedges match with outer depressions of the stem (black arrowhead). **G** *Niedenzuella multiglandulosa* cambium is continuous throughout the phloem wedge (Pw), evidenced by the formation of inclined cells of phloem and xylem (yellow dotted lines and arrowhead). **H** *M. sepium* showing the formation of xylem and phloem perpendicularly to the previous tissues formed in the stem (yellow dotted lines and arrowhead), evidencing that the cambium is continuous, i.e., delineates the entire phloem wedge (Pw). **I** *Mascagnia divaricata* variant cambium produces under the phloem wedges (Pw) larger bands of non-lignified parenchyma (nlp) (arrowhead) than regular cambium. **J** *Peixotoa sericea* xylem under the phloem wedge (Pw) contains fewer vessels and more fibers, phloem wedges match with outer depressions of the stem (black arrowhead). **K** *Mascagnia sepium* parenchyma proliferation (pa) under the phloem wedges (Pw) proliferates (arrowhead) breaking up the inner xylem, stimulating proliferation also of the pith (p), in a continuum. Scale bars **A**–**C**, **I**–**J** = 1 mm, **D** = 5 mm, **E**, **F** = 200 µm, **G**, **H** = 400 µ, **K** = 500 µm

All phloem wedges of Malpighiaceae have a continuous cambium in at least one stage of their development (Fig. 4). While in some species this configuration is kept as their final form (Fig. 5A–E), others progress to other stem anatomies (Fig. 5F–H). Anatomically, all species with phloem wedges start their secondary growth with a regular, continuous cambium producing tissues equally across its girth (Figs. 4, 6A). However, sooner or later, in certain cambial regions, the cambium switches its activity and starts producing less secondary xylem and more secondary phloem, initially forming shallow arcs or invaginations (Fig. 6B) and then progressing into phloem wedges (Fig. 6C, D). Because the amount of phloem produced does not always keep up with the amount of xylem produced by the regular cambium, the external depressions mentioned above are formed (as seen in Figs. 3, 6F, J).

It is common in most genera and species that we studied that the number of phloem wedges increases in time, such as seen in *Mascagnia* (Fig. 5B). This phenomenon is evidenced by the co-occurrence of wedges of different depths within the stem (Figs. 5B and 6D, F). In some species, the variant cambium of the wedges produces very little xylem, and the wedges can deeply furrow the xylem (Figs. 5B and 6D, E, H, K).

Regardless of the depth or number of the phloem wedges, the cambium in species with this configuration remains continuous. The cambium continuity is evidenced by the persistent production of phloem and xylem from the variant cambia, which will generate phloem and xylem with a slightly different orientation. This is more evident in the secondary xylem, where new tissue is inclined or even perpendicular to the other cells in the stem (Fig. 6G, H).

Another remarkable aspect of the variant cambia that generate phloem wedges is that the xylem produced is not similar to that of the interwedges. In *Mascagnia* and species of *Stigmaphyllon*, the variant cambium produces xylem with much more non-lignified parenchyma than the regular cambium of the interwedge (Fig. 6I). In *Peixotoa* the variant cambium produces xylem with fewer vessels and more fibers (Fig. 6J).

**Fig. 7:**
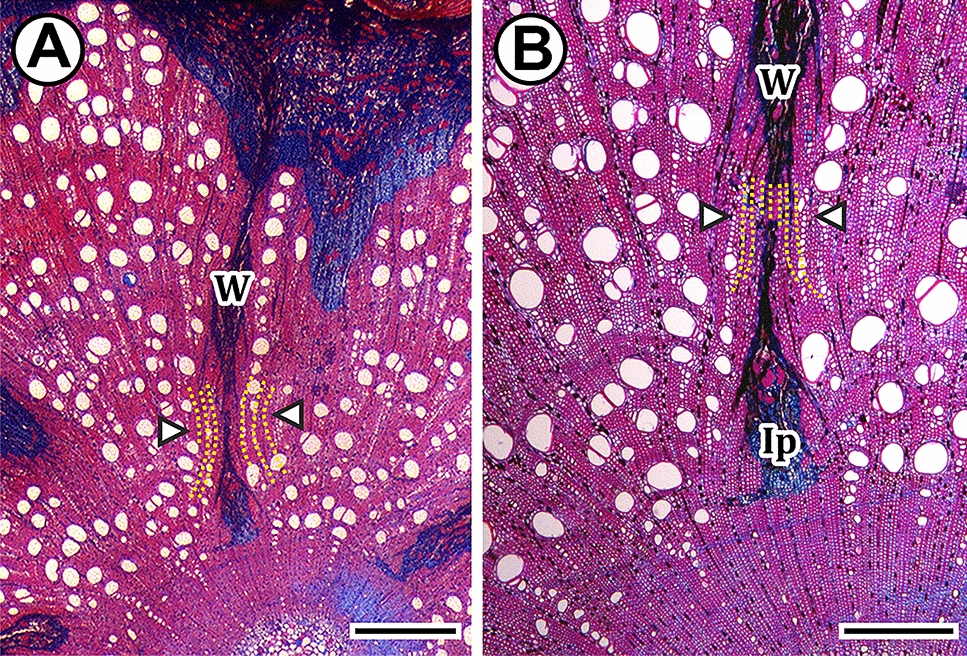
Light micrographs showing phloem wedges inclusion in cross-sections. All sections were double-stained in safranin and Astra-blue. **A**, **B** *Niedenzuella multiglandulosa.* **A** The inclusion of wedges portions is given by the mechanical pressure exerted by the adjacent wood at both sides of the phloem wedges (W) (yellow dotted lines and arrowheads). **B** Once the portion of the phloem wedge is included (Ip), the adjacent regions of the wedges are in contact (yellow dotted lines and arrowheads). Scale bars **A** = 1 mm, **B** = 500 µm

In thick stems of species of the Christianella clade, Carolus clade, *Diplopterys*, and some *Banisteriopsis* and *Mascagnia*, the non-lignified parenchyma, below the phloem wedges proliferates, inducing proliferation also of the pith in a continuum, anastomosing the deepest wedges in the stem, forming fissured stems. (Fig. 4A, ontogeny 1a, 6K). On the other hand, in *Niedenzuella*, the cambium is continuous throughout the plant ontogeny, but some portions of the phloem wedges get included by coalescence of the xylem on each side of the wedges. This inclusion form patches of interxylary phloem under the wedges (Fig. 7A, B), which maintain reminiscences of an active vascular cambium. The inclusion of phloem wedges is a result of mechanical pressure from both sides of the phloem wedge. The xylem encloses part of phloem wedges since wood production by the regular cambium is greater than that of the variant cambium. This differential production exerts pressure on both sides of the wedge including their innermost parts.

#### Phloem wedges with a discontinuous cambium form a stepwise pattern during their development (Ontogeny II)

**Fig. 8:**
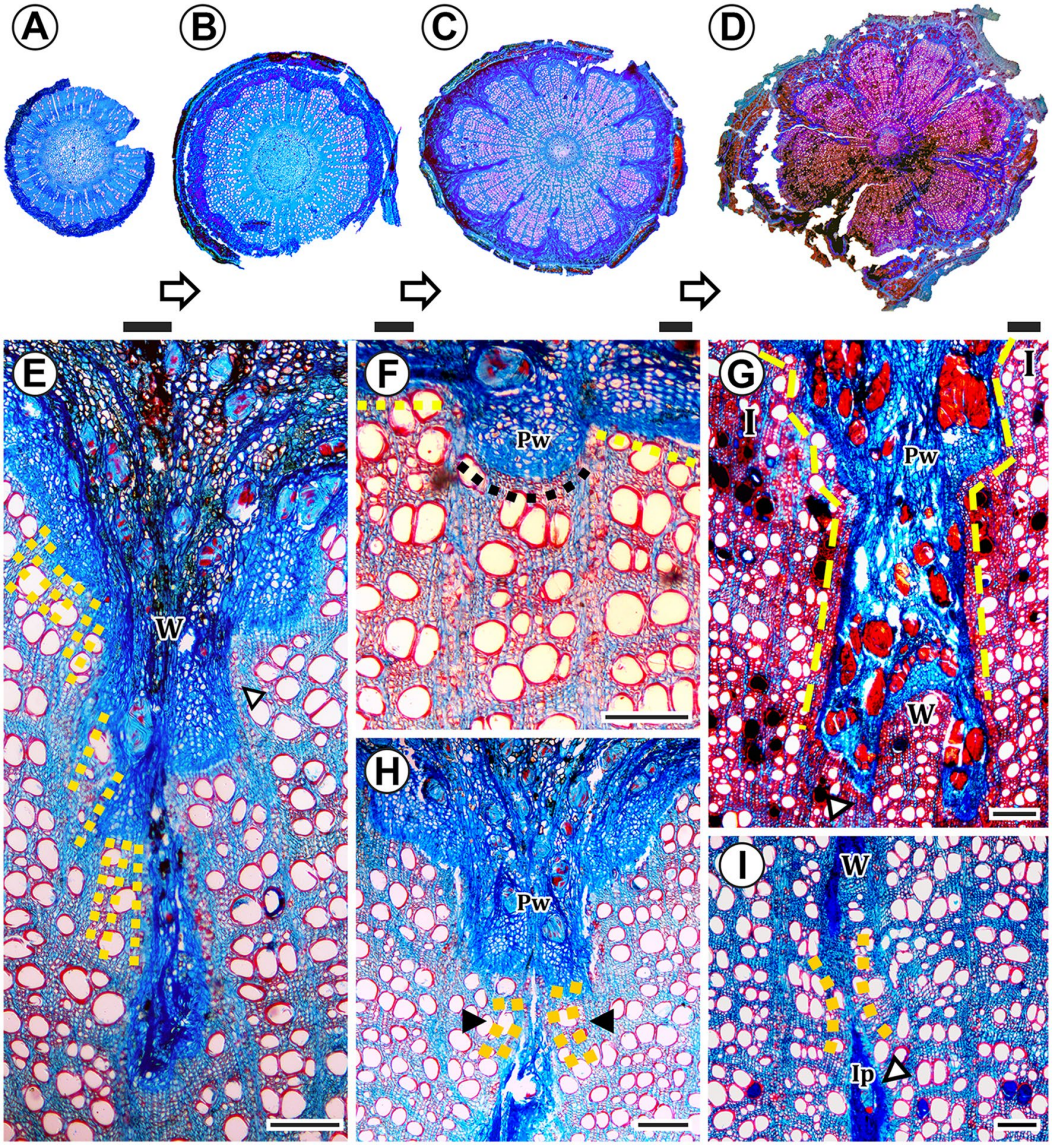
Light micrographs of stem cross-section showing phloem wedges formation with cambium disruption (Ontogeny II). All sections were double-stained in safranin and Astra-blue. **A–D** Development of phloem wedges in *Tristellateia greveana.* **A** Onset of development with a single, continuous cambium with regular activity. **B** Formation of shallow phloem invaginations, i.e., the onset of differential production between secondary xylem and phloem. **C** Shallow phloem arcs turn into phloem wedges. **D** Final architecture with well-formed phloem wedges and phloem inclusion. **E–I** *Tristellateia greveana.* **E** Onset of cambium disruption in the wedge region (W), evidenced by the formation of inclined cells of xylem (yellow dashed lines) and cells with a parallel course to those produced by regular cambium, limiting rays throughout wedges (arrowhead). **F** Since early stages it is possible to see the disruption between regular cambium (yellow dashed lines) and variant cambium (black dashed lines). **G** Phloem wedges with a stepwise pattern (yellow dashed lines). Note that the wood produced by the variant cambium in the wedge region (W) is like that produced by the regular cambium in the interwedge region (I). **H** The wood that flanks the phloem wedge (Pw) begins to exert pressure on both sides of the wedge (yellow dashed lines and black arrowheads). **I** Mechanical pressure eventually embeds a portion of the phloem wedge (Ip) within the wood (arrowhead), the adjacent regions of the wedges are in contact (yellow dashed lines), the interxylary phloem (Ip) has minute reminiscences of variant cambium. Scale bars **A–D** = 1 mm **E**–**I** 200 µm

This type of cambial variant was found exclusively in the paleotropical genus *Tristellateia*, from the Bunchosia clade (Fig. 4, ontogeny 2, Fig. 5G, H, Fig. 8). The secondary growth begins with the differentiation of a single and continuous cambium with regular activity (Fig. 8A), similarly to the previously discussed ontogeny. While the stem progresses with secondary growth, small phloem invaginations start to be formed by certain areas of the cambium which reduce the production of xylem and increase the production of phloem (Fig. 8B); these areas are alternate with leaf insertions. Later in development, the shallow phloem arcs acquire the shape of wedges (Fig. 8C, D). Although it can be perceived in much earlier stages (Fig. 8F), it is during this stage that cambium disruption is most evident (Fig. 8E). Disruptions are caused by a decrease or cessation of anticlinal divisions in the variant cambial region. Cambium continuity is lost, and the wedges become flanked by conspicuously wide rays, known as limiting rays (Fig. 8E).

Phloem wedges widen up towards the regular cambium region (Fig. 8E, G). The regular cambium near the original variant cambium switch from a regular to a variant activity, resulting in a stepwise pattern (Figs. 1C, D, 8E, G). The variant cambial portions get disconnected from the regular cambial portions as phloem wedges widen up. These variant cambial portions remain on the bottom of the wedges (Fig. 8E), being still active and producing both secondary xylem and phloem.

In some wedges, similarly to what is seen in *Niedenzuella*, the secondary xylem that flanks the external most part of the wedge, begins to exert pressure from both sides, eventually encapsulating portions of the phloem wedge within the wood, forming islands of interxylary phloem (Fig. 8H, I). It means that higher production of wood by the regular cambium generates pressure on both sides of the wedge and mechanically includes it. As portions of phloem wedges are included by adjacent wood, the limiting rays merge. This phenomenon evidences that the inclusion of phloem results from mechanical pressure that the adjacent wood exerts on both sides of the wedge. The interxylary phloem patches retain reminiscences of variant cambium. The wood produced by the variant cambial portions seems to be qualitatively equal to that produced by the regular cambial portions.

## Discussion

### Phloem wedges have evolved multiple times within Malpighiaceae, exclusively in lianas

Stems furrowed by phloem wedges are one of the most common, widespread types of cambial variants in angiosperms [9, 10, 13]. They had been recorded within some of the richest neotropical liana lineages, such as Bignoniaceae and Sapindaceae [9, 10, 58, 59], and their formation is intimately related to an increase in stem flexibility, breakage prevention when twisting or bending, and injury repair [7, 14, 60–62]. In Malpighiaceae, phloem wedges are also the most common cambial variant type. Here, we elucidate that its evolution likely occurred at least 10 times independently and is correlated to the evolution of lianas (Fig. 1).

Phloem wedges are most common in the large clades known as tetrapteroids and stigmaphylloids being present in 6 independent lineages with 8 independent origins. Only in two instances, in the Hiraea and the Bunchosia clades, phloem wedges evolved outside of these two large clades. These two instances constitute the first record altogether of cambial variants outside the tetrapteroid and stigmaphylloid clades in Malpighiaceae. The Hiraea clade is sister to the tetrapteroids and stigmaphylloids clades, while Bunchosia clade is sister to tetrapteroids + stigmaphylloids + Hiraea clades (Fig. 1) and has one of the most species-rich genera in the family, the arboreal *Bunchosia* [63]. While *Hiraea* is a neotropical genus being more diverse in northwestern South America [5, 64], *Tristellateia* is the only paleotropical genus within the Bunchosia clade, exclusively distributed in continental Africa, Madagascar, and South-east Asia [65].

Previous reports on Malpighiaceae [12, 17, 21, 23, 27] have mentioned and described the presence, development, and structure of the phloem wedges in scattered members of the family. Jussieu [17] was perhaps the first to report lianas with deep phloem wedges in Malpighiaceae, while Schenck [21] later described in detail these wedges in species that at that time were under the genera *Tetrapterys, Heteropterys*, and *Peixotoa*. Studies on the role of cambial variants in injury repair have also mentioned the presence of phloem wedges in *Peixotoa glabra* and *Mascagnia psilophylla* (= *Callaeum psilophyllum*) [14]. Other authors described in detail the ontogeny of fissured stems, showing that the phloem wedges were a stage in their development [23, 27], which is here named ontogeny 1a (Fig. 4).

The presence and position of the phloem wedges in the stems of different lineages have been consistently shown to be non-random [27, 31, 66, 67]. In Malpighiaceae, the phloem wedges were always seen developing between leaf insertions; like in Bignoniaceae where phloem wedges are also alternate to the leaves [31, 66]. The fact that their presence is correlated to the leaf position reveals that they are intimately linked to auxin regulation, evidencing the delicate, intricate processes controlling the formation of cambial variants [10, 68].

### Two main ontogenies explain the evolution of phloem wedges

Although they have evolved multiple times independently, we were able to narrow down the ontogenies of phloem wedges in Malpighiaceae into 2 main types, those in which the phloem wedges maintain the continuity of the cambium and those where the wedges cause the dissection of the vascular cambium. Regarding their formation, despite the two different ontogenetic trajectories, both share the same early stages of development, with an initial regular growth followed by shallow arcs and then phloem wedges. The late stages of development are those that distinguish each trajectory, being, therefore, a case of peramorphosis heterochrony, with terminal additions of new stages in comparison to the ancestral anatomies [69]. Of all the species analyzed in this work, those stems with phloem wedges that maintain the cambium continuity were the most common, being present in 7 of the 8 lineages in which phloem wedges evolved. This implies that the anticlinal divisions of the variant cambium typically keep up with those of the regular cambium, preserving cambial continuity. This continuity in the cambium can be distinguished through the products of its divisions and their orientation, that is, as the cambium remains continuous, the products formed on the flanks of the phloem wedges are perpendicular to the tissues formed by the regular cambium.

On the other hand, in those stems where the cambium is discontinuous, the anticlinal divisions were never found in the variant cambium, refraining it to keep pace with the adjacent regions of regular growth, and leading to their inclusion in the inner part of the phloem wedges. This is evidenced by the production of secondary tissues by both the regular and variant cambia, where the tissues produced have similar orientations. The presence of phloem wedges with a discontinuous cambium is likely a synapomorphy of the species-rich *Tristellateia* genus. In other lineages such as Bignoniaceae or Leguminosae where the cambium loses its continuity, this process is also a result of the interruption in anticlinal divisions by the variant cambia [10, 31]. How these variant cambia lose the capacity of anticlinal division needs to be explored in gene-regulation studies in the future.

### The development of phloem wedges reflects modularity in the vascular cambium

Secondary xylem and secondary phloem are both derived ontogenetically from the same cell lineage, the vascular cambium. The differential production of secondary tissues by the variant cambial portions within phloem wedges could be interpreted as a case of modularity in the vascular cambium [32] since there is a concerted decrease in the production of secondary xylem and an increase in the production of secondary phloem. Simultaneously, certain portions of the cambium here named “regular” maintain in its initial activity, hence suggesting quasi-independence of both vascular cambium portions [58]. Both these regions, however, make up the entire organ functional. The presence of phloem wedges interrupting the xylem has long been shown to be a mechanism that increases the flexibility of these plants during their ontogenies [14].

Modularity in the processes controlling secondary growth helps promote different vascular configurations, arrangements, and amounts of secondary tissues. Modularity is moreover key to understanding how one part of an organism can change evolutionarily, maintaining the functionality of the entire structure [70]. In stem development, modularity on vascular cambium allows explaining the processes that lead to the development of more complex forms where the coordination of the different portions of the vascular cambium is fundamental. Here we find that phloem wedges are the first step leading to the evolution of even more complex anatomies in Malpighiaceae, such as interxylary phloem and fissured stems, therefore greatly contributing to the anatomical diversification of the family. Also, the selection of the functionality of this structure to climbing has likely operated in the evolution of this modular growth.

### Inclusion of phloem wedges (interxylary phloem)

This is the first report of included phloem wedges in the family, for the neotropical genera *Niedenzuella* of the Niedenzuella clade and the paleotropical *Tristellateia* of the Bunchosia clade. Although interxylary phloem in Malpighiaceae was previously known only in genus *Dicella*, the phloem strands are formed by a single cambium producing phloem to the outside and inside simultaneously at certain moments [20, 23], a completely different process.

A similar phenomenon seen in *Niedenzuella* and *Tristellateia* was described in Bignoniaceae, in the genus *Amphilophium* [31], but the mechanism of phloem wedges inclusion is not the same. In *Amphilophium*, the phloem is included because the cambium of the adjacent regions of the phloem wedges triggers the re-differentiation of axial parenchyma cells from the nonconducting phloem, enclosing part of the phloem wedge within newly formed wood. In later stages of development, new wedges may be formed in the same region of the cambium where the previous wedge arose [31].

On the contrary, in *Niedenzuella* and *Tristellateia* the differential production of cambium between the wedge and interwedge regions causes a higher production of secondary xylem in the interwedge region. The increase in this stiff tissue exerts a mechanical force on both sides of the wedges, that ends up including the wedges in the wood, forming small patches of interxylary phloem. In the variant regions where phloem wedges were included, new wedges continued to be formed.

### Fissured stems

Fissured stems are the most emblematic type of cambial variant of Malpighiaceae, with similar anatomies only rarely seen in other lineages such as Convolvulaceae (*Distimake*) and Passifloraceae (*Passiflora*) and likely also involving an earlier formation of phloem wedges [71]. Hence, our work reveals that the evolution of one cambial variant can be a step to the evolution of even more complex cambial variants, boosting the anatomical diversity present in their families. Fissured stems are formed by the combination of phloem wedges and non-lignified parenchyma next to the wedges that proliferated and dissect the entire structure [23, 27].

## Conclusions

Phloem wedges are a common cambial variant type of Malpighiaceae, evolving at least 10 times independently in 8 of the main lineages within the family. Their evolution is exclusive in lianas, being lost in self-supporting groups nested within lianas lineages. Two main ontogenies explain the formation of phloem wedges, one in which the cambium keeps its continuity and one in which the interruption of anticlinal divisions causes the discontinuity of the variant cambium. In two genera, the neotropical *Niedenzuella* and paleotropical *Tristellateia*, the regular portions can push the edge of the phloem wedges causing their inclusion, forming a novel type of interxylary phloem not previously recorded for the family, and likely synapomorphic for these genera. Phloem wedges are also an intermediate stage for all the lineages with fissured stems in Malpighiaceae, suggesting that complex cambial variants can result from initially more simple types. Further studies are being carried out to explore whether the evolution of these cambial variants can have boosted the diversification of the lianescent lineages in Malpighiaceae.

## Supplementary Information

**Additional file 1: List S1.** Species that were collected in search of cambial variants, for this study. Data collection is provided. **Table S1** List of taxa included in the phylogenetic studies along with its GenBank accession numbers.

**Additional file 2: Figure S1.** Maximum clade credibility tree (MCC) with divergence time estimates for Malpighiaceae.

**Additional file 3: Table S3.1.** Character data set used for the ancestral state reconstruction

**Additional file 4: Table S4.1** Hypothesis test comparing the log-likelihood and Akaike score (AIC) between equal rates (ER), and all rates different (ARD) transition models. **Table S4.2** Stochastic character map results. Changes between each character state along the Malpighiaceae phylogeny. **Table S4.3** Pagel’s 1994 Test of Correlated Evolution Results. **Table S4.4** Results from Pagel’s λ and Blomberg’s K tests for phylogenetic signal.

## Data Availability

All data generated or analyzed during this study are included in this published article [and its supplementary information files].
