# Supplementary material for: Phloem wedges in Malpighiaceae: origin, structure, diversification, and systematic relevance
Source: EvoDevo. 2022 Apr 28;13:11. doi: 10.1186/s13227-022-00196-3 (PMC9052467; doi:10.1186/s13227-022-00196-3)
Supplement: Supplementary file 1 — Additional file 1: List S1. Species that were collected in search of cambial variants, for this study. Data collection is provided. Table S1 List of taxa included in the phylogenetic studies along with its GenBank accession numbers. [file 13227_2022_196_MOESM1_ESM.docx]

# **Additional file 1**

**Article title**: Phloem wedges in Malpighiaceae: origin, structure, diversification, and systematic relevance

**Authors:** Quintanar-Castillo A and Pace MR, 2022

## **List S1.** Species that were collected in search of cambial variants. We provide taxa name, collector, and locality. Vouchers of all species were deposited in herbaria indicated after the other information of each plant (abbreviations following Thiers [34]); duplicates indicated when present.

***Acmanthera latifolia*** Griseb. Yale 40099 Photographed at the Madison Forest Products Laboratory***. Acridocarpus chevalieri*** Sprague, *Jongkind* 4394, Ivory Coast, Bouflé, Parc National de la Marahoué, WAG. ***A***. ***excelsus*** A. Juss., *Pace* 1035, 1038, Madagascar, Fianarantsoa, Ranohira, Ihorombe, TAN, duplicate at MO and P. ***A. longifolius*** (G.Don) Hook.f., *Bos* 5596, Cameroon, Kribi, WAG. ***A. macrocalyx*** Engl., *Wieringa* 4376, Gabon, Koubougue, 13km NE of Libreville, WAG; *Elad* 565, Cameroon South Province, Ebom, Muila, WAG. ***A. plagiopterus*** Guill. & Perr., *Breteler* 5468, Liberia, Nimba Mt., WAG. ***Aenigmatanthera lasiandra*** (A.Juss.) W.R.Anderson, *Pace* 463, Brazil, Mato Grosso do Sul, Rio, SPF. ***Alicia anisopetala*** (A.Juss.) W.R.Anderson, *Pace* 198, 199, 200, Brazil, Mato Grosso do Sul, Corumbá, SPF, duplicates at SP, MICH, and US; *Pace* 501, Brazil, Mato Grosso do Sul, SPF. ***A. macrodista*** (Triana & Planch) Griseb., *Acevedo* 1601, Peru, Loreto,Cano Eicagua, Sapuena, Requena, U, duplicate at NY. ***Amorimia rigida*** (A.Juss.) W.R.Anderson, *Pace* 350, Brazil, Bahia, Barro Preto, SPF. ***Aspicarpa harleyi*** W.R.Anderson, *Almeida* s.n., Brazil, Bahia, Caeteté, SPF. ***Aspidopterys nutans*** (Roxb. ex DC.) A.Juss., *Maxwell* 89-333, Thailand, Chang Mai, Muang district, L. ***Banisteriopsis angustifolia*** (A.Juss.) B.Gates, *Pac*e 306, Brazil, Minas Gerais, SPF. ***B. argyrophylla*** (A.Juss.) B.Gates, *Pace* 272, 294, Brazil, Goiás, SPF, duplicates at K, US, MICH; *Pace* 321, Brazil, Minas Gerais, SPF. ***B. campestris*** (A.Juss.) Little, *Pace* 293, Brazil, Goiás, Alto Paraíso de Goiás, SPF; *Pace* 295, 299, Brazil, Goiás, Pirenópolis, SPF; *Pace* 320, Brazil, Minas Gerais, Datas,mSPF. ***B. gardneriana*** (A.Juss.) W.R.Anderson & B.Gates, *Pace* 328, Brazil, Minas Gerais, Diamantina, SPF; *Ceccantini* 3724, Brazil, Tocantins, Almas, SPF. ***B. laevifolia*** (A.Juss.) B.Gates, *Pace* 309, Brazil, Minas Gerais, Diamantina, SPF, duplicate at K; *Pace* 323, Brazil, Minas Gerais, Datas, SPF; duplicate at US; *Ceccantini* 3599, 3600, Brazil, Minas Gerais, Santana do Riacho, SPF. ***B. malifolia*** (Nees & Mart.) B.Gates*, Pace* 268, 269, Brazil, Goiás, Alto Paraíso de Goiás, SPF, duplicate at K; *Pace* 296, 297, 298, Brazil, Goiás, Pirenópolis, SPF, duplicate at US; *Pace* 326, Brazil, Minas Gerais, Diamantina, SPF. ***B. megaphylla*** (A. Juss.) B. Gates, *Pace* 606, Brazil, Mato Grosso do Sul, Aquidauana.***B. muricata*** (Cav.) Cuatrec., *Pace* 330, Brazil, Minas Gerais, São Gonçalo do Rio Preto, SPF, duplicate at K. ***B. nummifera*** (A. Juss.) B. Gates**,** *Pace* 258, Brazil, Pernambuco, Igarassú, SPF; *Pace* 337, Brazil, Bahia, Ilhéus, SPF; *Ceccantini* 3748, Brazil, Tocantins, Almas, SPF. ***B. oxyclada*** (A.Juss.) B.Gates, *Pace* 398Brazil, Bahia, Maracás, SPF, duplicate at K, CEPEC. ***B. stellaris*** (Griseb.) B.Gates, *Pace* 276, 277, Brazil, Goiás, Alto Paraíso de Goiás, SPF, duplicate at US, MICH; *Pace* 280, 282, 284, Brazil, Goiás, Alto Paraíso de Goiás, SPF; *Pace* 289, Brazil, Goiás, Alto Paraíso de Goiás; *Pace* 310, Brazil, Minas Gerais, Diamantina, SPF; *Pace* 333, Brazil, Minas Gerais, São Gonçalo do Rio Preto, SPF. ***Barnebya harley*** W.R.Anderson & B.Gates, *Almeida* s.n., Brazil, Bahia, Itatim, SPF. ***Bronwenia peckorii*** W.R.Anderson & C.Davis, *Kubo* 188, Brazil, Bahia, Araçuaí, SPF. ***Bunchosia argentea*** (Jacq.) DC., *Breteler* 4610, ca. 70km from Barinas along road to San Cristóbal, Barinas, Venezuela, voucher at WAG. ***B. glandulifera*** (Jacq.) Kunth, *Pace* 266, 267, Brazil, Pernambuco, Recife, SPF, duplicates at CEPEC, MICH. ***B. maritima*** (Vell.) J.F. Macbr., *Reitz & Klein* 3596, Brazil, Ibarama, SPF. ***B. montana*** A.Juss., *Pace* 416, 418, Mexico, Oaxaca, Magdalena Ocotlán, SPF. ***B. polystachia*** (Andrews) DC. *Pace* 692, Costa Rica, Reserva Ecologica La Selva, US. ***Byrsonima coccolobifolia*** Kunth, *Pace* 667, 680, Brazil, São Paulo, Pratânia, US, duplicate at SPF. ***B. cydoniifolia*** A.Juss., *Pace* 203, 204, Brazil, Mato Grosso do Sul, Corumbá, SPF, duplicate at CEPEC; *Pace* 324, Brazil, Minas Gerais, Datas, SPF; *Lovo* 366, Brazil, Minas Gerais, Diamantina, SPF. ***B. intermedia*** A.Juss., *Pace* 152, Brazil, São Paulo, Botucatu, SPF. ***B. pachyphylla*** A.Juss., *Pace* 281, 288, Brazil, Goiás, Alto Paraíso de Goiás, SPF, duplicate at CEPEC. ***B. poeppigiana*** A.Juss.,*Pace* 528, Peru, Loreto, Iquitos, SPF. ***B. sericea*** DC., *Pace* 251, Brazil, Pernambuco, Igarassú, SPF, duplicates at CEPEC, MICH; *Pace* 271, Brazil, Goiás, Alto Paraíso de Goiás, SPF, duplicate at CEPEC. ***B. stipulina*** J.F. Macbr., *Pace* 522, 524, Peru, Loreto, Iquitos, SPF. ***B. subterranea*** Brade & Markgr., *Ceccantini* 3782, Brazil, Tocantins, Almas, SPF. ***B. verbascifolia*** (L.) DC., *Pace* 301, Brazil, Goiás, Alto Paraíso de Goiás, SPF; *Pace* 682, Brazil, São Paulo, Pratânia, US, duplicate at SPF. ***B. viminifolia*** A.Juss., *Pace* 274, 275, Brazil, Goiás, Alto Paraíso de Goiás, SPF, duplicates at CEPEC, K, MICH; *Pace 302*, Brazil, Goiás, Alto Paraíso de Goiás, SPF, duplicate at CEPEC. ***Calcicola parvifolia*** (A. Juss.) W.R. Anderson & C. Davis, *Pace* 897, Mexico, Oaxaca, Coixtlahuaca, MEXU, duplicates at SPF, US, MO, MICH; *Pace* 1224, Mexico, Puebla, Zapotitlán de Salinas, MEXU. ***Callaeum antifebrile*** (Ruiz ex Griseb.) D.M.Johnson, *Pace* 220, 433, Brazil, Rio de Janeiro, SPF, duplicate at CEPEC. ***C. macropterum*** (DC.) D.M.Johnson, *Pace* 999, Mexico, Aguascalientes, Calvillo, MEXU. ***C. malpighioides*** (Turcz.) D.M.Johnson, *Acevedo-Rodríguez* 16442, Mexico, Chiapas, Palenque, US ***C. psilophyllum*** (A.Juss.) D.M.Johnson, *Pace* 542, 561, Argentina, Buenos Aires, La Plata, SPF. ***Camarea axillaris*** A.St.-Hil., *Lovo* 453, Brazil, Minas Gerais, Diamantina. ***Carolus chasei*** (W.R.Anderson) W.R. Anderson, *Pace* 400, Brazil, Bahia, Maracás, SPF, duplicate at K, MICH. ***Christianella mesoamericana*** (W.R. Anderson) W.R. Anderson**,** *Acevedo-Rodríguez* 16389, Mexico, Chiapas, US. ***C. multiglandulosa*** (Nied.) W.R. Anderson, *Pace* 640, 643, Brazil. São Paulo, Assis. ***Cordobia argentea*** (Griseb.) Nied., *Pace* 1188, Argentina, Córdoba, Minas, Cruz del Eje, MEXU, duplicate at CTES. ***Dicella nucifera*** Chodat, *Pace* 147, Brazil, Jardim Botânico São Paulo, Nova Odessa, SPF, duplicate at CEPEC. ***Diplopterys amplectens*** (B.Gates) W.R.Anderson & C.Davis, *Pace* 469, Brazil, Mato Grosso do Sul, Rio Verde de Mato Grosso, SPF. ***D. lutea*** (Griseb.) W.R.Anderson & C.Davis, *Pace* 485, Brazil, Mato Grosso do Sul, Miranda, SPF; *Pace* 487, Brazil, Mato Grosso do Sul, Porto Murtinho, SPF; *Ceccantini* 3794, Brazil, Tocantins, Almas, SPF. ***D. patula*** (B.Gates) W.R.Anderson & C.Davis, *Pace* 370, Brazil, Bahia, Guararema, SPF; *Pace* 380, Brazil, Bahia, Boa Nova, SPF, duplicates at K, MICH, CEPEC. ***D. pubipetala*** (A.Juss.) W.R.Anderson & C.Davis, *Pace* 292, Brazil, Goiás, Alto Paraíso de Goiás, SPF, duplicate at MICH; *Pace* 394, Brazil, Bahia, Boa Nova, SPF, duplicates at K, CEPEC; *Pace* 451, Brazil, Mato Grosso do Sul, Corguinho, SPF; *Pace* 467, 468, Brazil, Mato Grosso do Sul, Rio Verde de Mato Grosso, SPF. ***Echinopterys eglandulosa*** (A. Juss) Small *Acevedo-Rodríguez* 16222*,* Mexico, Oaxaca, Tonalá, US; *Pace* 1008, Mexico, Aguascalientes, Calvillo, MEXU, duplicates at SPF and US; *Pace* 1123, Mexico, Puebla, Zapotitlán de Salinas, MEXU. ***E. setosa*** Brandegee, *Pace* 949, 952, 952, Mexico, Coahuila, Cuatro Ciénegas, MEXU, duplicates at SPF and US*.* ***Excentradenia adenophora*** (Sandwith) W.R. Anderson, *Lindem/Heyde* 130, 652, no more info in the wood collection, voucher at U. ***E. propinqua*** (W.R. Anderson) W.R. Anderson, *Linderman+ stud*. 51, no more info in the wood collection, voucher at U. ***Flabellaria paniculate*** Cav., *Versteegh* 494, Ivory Coast, WAG; *Bos* 3509, Cameroon, seashore 9km n of Kribi, WAG; *Koning* 6751, Ivory Coast, Abidjan, WAG. ***Gaudichaudia albida*** Schltdl. & Cham., *Pace* 419, Mexico, Oaxaca, Ocotlán, SPF; *Pace* 423, Mexico, Veracruz, La Mancha, SPF. ***Glicophyllum cardiophyllum* (Nied.) R.F. Almeida**, *Almeida* s.n., Brazil, Bahia, Rio de Contas. ***G. microphyllum* (Nied**.) **R. F. Alemida**, *Pace* 312, 313, Brazil, Minas Gerais, Diamantina, SPF; *Pace* 331, Brazil, Minas Gerais, São Gonçalo do Rio Preto, SPF. ***Heladena multiflora*** (Hook & Arn). Nied. *Nejapa* 3, 4, Argentina, Corrientes, San Cosme, MEXU. ***Heteropterys arenaria*** Markgr., *Pace* 385, Brazil, Bahia, Boa Nova, SPF. ***H. bicolor*** A.Juss., *Pace* 361, Brazil, Bahia, Teimoso, SPF. ***H. brachiata*** (L.) DC., *Pace* 406, Mexico, Veracruz, Jalcomulco, SPF. ***H. bullata*** Amorim, *Pace* 347, Brazil, Bahia, Ilhéus, SPF; *Pace* 381, Brazil, Bahia, Boa Nova, SPF. ***H. campestris*** A.Juss., *Pace* 291, Brazil, Goiás, Alto Paraíso de Goiás; *Pace* 670, Brazil, São Paulo, Pratânia. ***H. chrysophylla*** (Lam.) Kunth, *Somner* 1627, 1628, Brazil, Rio de Janeiro, Tinguá, RBR; *Pace* 616, Brazil, São Paulo, São Sebastião, US. ***H. coleoptera*** A. Juss., *Pace* 689, Brazil, São Paulo, Bertioga, SPF. ***H. cordifolia*** Moric., *Pace* 256, Brazil, Pernambuco, Igarassú, SPF; *Pace* 343, Brazil, Bahia, Ilhéus, SPF. ***H. eglandulosa*** A.Juss., *Pace* 384Brazil, Bahia, Boa Nova, SPF. ***H. glabra*** Hook. & Arn., *Pace* 144, Brazil, São Paulo, Nova Odessa, SPF, duplicate at CEPEC. ***H. imperata*** Amorim, *Pace* 335, 341, Brazil, Bahia, Ilhéus, SPF. ***H. intermedia*** A.Juss., *Pace* 158, Brazil, São Paulo, SPF; *Pace* 382, Brazil, Bahia, Boa Nova, SPF; *Pace* 531, Brazil, São Paulo, Bertioga, SPF. ***H. laurifolia*** (L.) A.Juss., *Pace* 712, Costa Rica, Sarapiquí, Reserva Biológica La Selva, US; *Pace* 754, Mexico, Veracruz, Arroyo de Oro, MEXU, duplicate at US. ***H. leona*** (Cav.) Exell, *Wit* s.n., Liberia, WAG; *Versteegh* 634, Ivory Coast, WAG; *Breteler* 1621, Cameroon, Nyong River, WAG. ***H. nitida*** DC., *Pace* 163, 167, Brazil, São Paulo, Santo André, SPF; *Pace* 226, 233, Brazil, Rio de Janeiro, Reserva Biológica de Poço das Antas, SPF, *Pace* 621, Brazil, São Paulo, São Sebastião, US. ***H. nordestina*** Amorim, *Pace* 360, Brazil, Pernambuco, Igarassú, SPF. ***H. panamensis*** Cuatrec. & Croat, *Pace* 707, 708, Costa Rica, Sarapiquí, Reserva Biológica La Selva, US. ***H. patens*** (Griseb.) A.Juss., *Pace* 168, Brazil, São Paulo, Santo André, SPF. ***H. pteropetala*** A.Juss., *Pace* 270, Brazil, Goiás, Alto Paraíso de Goiás, SPF, duplicate at MICH; *Pace* 273, Brazil, Goiás, Alto Paraíso de Goiás, SPF, duplicate at K; *Pace* 278, , Brazil, Goiás, Alto Paraíso de Goiás, SPF. ***H. rhopalifolia*** A.Juss., *Ceccantini* 3779, 3780, 3781, Brazil, Tocantins, Almas, SPF. ***H. thyrsoidea*** A. Juss., *Pace* 164, 165, Brazil, São Paulo, Santo André, SPF. ***H. trichanthera*** A.Juss., *Pace* 395, Brazil, Bahia, Boa Nova, SPF. ***Hiptage benghalensis*** (L.) Kurz, *Pace* 434, Brazil, Rio de Janeiro, Arboreto do Jardim Botânico do Rio de Janeiro, SPF, duplicate at CEPEC. ***H. laurifolia*** A.Juss., Koloniaal Museum, Haarlem 1507-99, no more info in the wood collection, L. ***Hiraea bullata*** W.R.Anderson, *Pace* 351, Brazil, Bahia, Barro Preto, SPF. ***H. fagifolia*** (DC.) A. Juss., *Pace* 471, Brazil, Mato Grosso do Sul, Dourados, SPF. ***Janusia guaranitica*** (A.St.-Hil.) A. Juss., *Pace* 435, Brazil, São Paulo, SPF, duplicate at CEPEC; *Pace* 1166, Argentina, Córdoba, Cruz del Eje, MEXU, duplicate at CTES. ***J. schwannioides*** W.R. Anderson, *Pace* 391, Brazil, Bahia, Boa Nova, SPF. ***Jubelina wilburii*** W.R. Anderson, *Pace* 711, Costa Rica, Sarapiquí, Reserva Biológica La Selva, US. ***Lasiocarpus salicifolius*** Liebm., *Pace* 872, 873, México, Oaxaca, Santo Domingo Tonalá, MEXU. ***Lophopterys euryptera*** Sandwith, no info in the wood collection, voucher at U. ***Malpighia glabra*** L**.,** *Pace* 140, Brazil, São Paulo, Nova Odessa, SPF; *Pace* 424, Mexico, Veracruz, La Mancha, SPF. *Pace* 436, Brazil, São Paulo, SPF. ***M. mexicana*** A.Juss., *Pace* 413, 414, 417, Mexico, Oaxaca, Magdalena Ocotlán, SPF. ***Malpighiodes bracteosa****,* (Griseb.) W.R. Anderson, *Marinho* 1354, Brazil, Manaus, Amazonas, CEPEC. ***Mascagnia cordifolia*** (A.Juss.) Griseb**.**, *Pace* 359, Brazil, Bahia, Jussari, CEPEC. ***M. divaricata*** (Kunth) Nied., *Pace* 470, Brazil, Mato Grosso do Sul, Dourados, SPF. ***M. sepium*** (A.Juss.) Griseb., *Pace* 79, Brazil, São Paulo, SPF. ***Mcvaughia* *sergipana***, Monteiro, *Almeida* s.n., Brazil, Sergipe, Pirambu, SPF. ***Mezia mariposa*** W.R.Anderson, *Pace* 72, Brazil, Amazonas, Reserva Adolpho Ducke, SPF. ***Niedenzuella acutifolia*** (Cav.) W.R. Anderson, *Pace* 342, 344, Brazil, Bahia, Ilhéus, SPF. ***N. multiglandulosa*** (A. Juss.) W.R. Anderson, *Pace* 262, Brazil, Pernambuco, Igarassú, SPF. ***Peixotoa adenopoda*** C.E. Anderson, *Pace* 387, 389, Brazil, Bahia, Boa Nova, SPF, duplicates at K, US, MICH, CEPEC; *Pace* 399, Brazil, Bahia, Maracás, SPF. ***P. glabra*** A. Juss., *Pace* 308, 311, Brazil, Minas Gerais, Diamantina, SPF, duplicate at K. ***P. goiana*** C.E. Anderson, *Pace* 300, Brazil, Goiás, Pirenópolis, SPF. ***P. leptoclada*** A. Juss., *Pace* 604, Brazil, Mato Grosso do Sul, Aquidauana, SPF, duplicate at US. ***P. magnifica*** C.E. Anderson, *Pace* 465, Brazil, Mato Grosso do Sul, Rio Verde de Mato Grosso, SPF. ***P. sericea*** C.E. Anderson, *Pace* 346, 349, Brazil, Bahia, Ilhéus, SPF, duplicate at CEPEC. ***P. tomentosa*** A. Juss., Brazil. Minas Gerais, Diamantina, ***Philgamia glabrifolia*** Arènes, *Pace* 1011, 1013, Madagascar, Antananarivo, Antsirabe II, TAN, duplicate at MO. ***Pterandra pyroidea*** A.Juss. *Amorim* 8384, Brazil, Brasília, Area de Proteção Ambiental (APA) Cabeça de Veado, CEPEC. ***Ptilochaeta nudipes*** Griseb., *Pace* 591, Brazil, Mato Grosso do Sul, Corumbá, COR. ***Spachea elegans*** (G.Mey.) A.Juss., *Pace* 704, Costa Rica, Heredia, Reserva Biológica La Selva, US. ***S. tricharpa*** A. Juss., *Ll. Williams* 2243, Peru, Loreto, Caballo-Cocha, F. ***Sphedamnocarpus andersonii*** C.E. Anderson, *Pace* 1039*,* 1041, Madagascar, Fianarantsoa, Ihorombe, TAN, duplicates at MO and P. ***Stigmaphyllon acuminatum*** A.Juss., *Pace* 377, Brazil, Bahia, Dario Meira, SPF, duplicate at CEPEC. ***S. blanchetii*** C.E. Anderson, *Pace* 239, Brazil, Pernambuco, Igarassú, SPF. ***S. bonariense*** (Hook. & Arn.) C.E. Anderson, *Pace* 541, 560, Argentina, Buenos Aires, Isla Martín García, SPF. ***S. calcaratum*** N.E. Br., *Pace* 1156*,* Argentina, Santa Fé, Colastiné Sur, MEXU, duplicate at CTES. ***S. cavernulosum*** C.E. Anderson**,** *Pace* 357, Brazil, Bahia, Jussari, SPF. ***S. ciliatum*** (Lam.) A.Juss., *Pace* 338, Brazil, Bahia, Ilhéus, SPF, duplicate at CEPEC. ***S. cordatum*** Rose ex Donn.Sm., *Pace* 142, Brazil, São Paulo, Nova Odessa, SPF. ***S. ellipticum*** (Kunth) A.Juss., *Pace* 403, Mexico, Veracruz, Jalcomulco, SPF; *Pace* 781, México, Veracruz, Puente Nacional, US. ***S. florosum*** C. Anderson, *Pace* 457, Peru, San Martín, Lamas, SPF. ***S. lindenianum*** A. Juss., *Pace* 703, Costa Rica, Heredia, Reserva Biológica La Selva, SPF. ***S. macropodum*** A.Juss*., Pace* 352, Brazil, Bahia, Olivença, SPF, duplicate at CEPEC; *Pace* 371, Brazil, Bahia, Guararema, SPF. ***S. perforatum*** (Cham.) Miers, Brazil, São Paulo, Assis, US, duplicate at SPF. ***S. retusum*** Griseb. & Oerst., *Pace* 808, 809, Mexico, Veracruz, Nuevo Poblado Villa Hermosa, MEXU, duplicate at SPF and CEPEC; *Pace* 832, Mexico, Chiapas, Malpaso, MEXU, duplicate at SPF. ***S. sinuatum*** (DC.) Adr. Juss., *Pace* 516, Peru, Loreto, Iquitos, at SPF. ***S. tomentosum*** A.Juss., *Pace* 180, 186, 187, Brazil, São Paulo, São Sebastião, SPF, duplicates at US, MO, CEPEC; *Pace* 236, Brazil, Rio de Janeiro, Silva Jardim, SPF; *Pace* 378, Brazil, Bahia, Dario Meira, SPF, duplicates at CEPEC, MICH. ***Tetrapterys* *crispa*** A.Juss., *Pace* 362Brazil, Bahia, Teimoso, SPF, duplicate at CEPEC. ***T. heterophylla*** (Griseb,) W.R. Anderson, *Pace* 893, Mexico, Oaxaca, San Miguel del Puerto, MEXU. ***T. longibracteata*** A.Juss., *Almeida* s.n., Brasil. ***T. mucronata*** Cav., *Pace* 248, Brazil, Pernambuco, Igarassú, SPF. ***T. phlomoides*** (Spreng.) Nied.,*Pace* 235, Brazil, Rio de Janeiro, Silva Jardim, SPF; *Pace* 345, Brazil, Bahia, Ilhéus, SPF, duplicate at CEPEC. ***T. schiedeana*** Schltdl. & Cham., *Pace* 402, Mexico, Veracruz, Jalcomulco, SPF; *Pace* 783, Mexico, Veracruz, Puente Nacional, MEXU. ***Thryallis brachystachys*** Lindl., *Almeida* s.n., Brazil, Minas Gerais, Jequitinhonha, HUEFS. ***Triaspis odorata*** (Willd.) A.Juss., *Jongkind* 1944, Ghana, Krobo hill, WAG, duplicate at MO. ***Tricomaria usillo*** Hook. & Arn., *Pace* 1181, 1197, 1198, Argentina, Córdoba, Cruz del Eje, MEXU, duplicate at CTES. ***Tristellateia australasiae*** A. Rich. *Fujii, Noshiro, Suzuki, Sugawa & Takahashi 15089,* Japón, Okinawa Yaeyama Gun, Pref. TUS duplicate at TWTw. ***T. grevenana*** Baill*, Pace* 1086, 1089, 1091, Madagascar, Toliara, Atsimo-Andrefana, MEXU. ***Verrucularia glaucophylla*** A.Juss., *Almeida* s.n., Brazil, Bahia, Rio de Contas.

## **Table S1.** GenBank accession numbers for all markers and species used in the phylogenetic analysis.

|  | Plastid | | | Nuclear |
| --- | --- | --- | --- | --- |
|  | *matK* | *ndhF* | *rbcL* | PHYC |
| **Malpighiales** |  |  |  |  |
| **Malpighiaceae** |  |  |  |  |
| *Acmanthera latifolia* (Juss.) Griseb*.* | AF344524 | AF351013 | AF344454 | AF500528 |
| *Acridocarpus chevalieri* Sprague | HQ247175 | AY137247 | HQ247417 | AY499050 |
| *Acridocarpus excelsus* A.Juss*.* | HQ247176 | AY137248 | HQ247418 | AY499051 |
| *Acridocarpus macrocalyx* Engl. | HQ247177 | AF351017 | HQ247419 | AF500532 |
| *Adelphia hiraea* (Gaertn.) W.R.Anderson | AF344566 | AF351059 | AF344498 | AF500563 |
| *Aenigmatanthera lasiandra* (A.Juss.) W.R.Anderson | HQ247185 | HQ246738 | HQ247429 | HQ246952 |
| *Alicia anisopetala* (A.Juss.) W.R.Anderson | AF344562 | AF351053 | AF344494 | AF500557 |
| *Amorimia amazonica* (Nied.) W.R.Anderson | HQ247186 | HQ246739 | HQ247430 | HQ246953 |
| *Amorimia rigida* (A.Juss.) W.R.Anderson | HQ247190 | AY499101 | HQ247433 | AY499075 |
| *Aspicarpa brevipes* (Sessé ex DC.) W.R.Anderson | AF344526 | AF351082 | AF344456 | AF500581 |
| *Aspicarpa harleyi* W.R.Anderson | HQ247192 | HQ246744 | HQ247434 | HQ246958 |
| *Aspicarpa pulchella* (Griseb.) O'Donell & Lourteig | AF344528 | AF351026 | AF344458 | HQ246960 |
| *Aspicarpa sericea* Griseb*.* | HQ247194 | HQ246746 | HQ247435 | HQ246961 |
| *Aspidopterys* sp | AF344529 | AF351019 | AF344459 | AF436796 |
| *Aspidopterys tomentosa* (Blume) A.Juss*.* | HQ247196 | HQ246749 | HQ247437 | HQ246964 |
| *Banisteriopsis angustifolia* (A.Juss.) B.Gates | HQ247198 | HQ246751 |  | HQ246967 |
| *Banisteriopsis argyrophylla* (A.Juss.) B.Gates | HQ247199 | HQ246752 | HQ247439 | HQ246968 |
| *Banisteriopsis caapi* (Spruce ex Griseb.) Morton | HQ247200 | HQ246753 | HQ247440 | HQ246969 |
| *Banisteriopsis laevifolia* (A.Juss.) B.Gates | HQ247205 | HQ246757 | HQ247441 | HQ246974 |
| *Banisteriopsis muricata* (Cav.) Cuatrec. | HQ247208 | HQ246760 | HQ247443 | HQ246977 |
| *Banisteriopsis nummifera* (A.Juss.) B.Gates | HQ247209 | HQ246761 | HQ247444 | HQ246978 |
| *Barnebya dispar* (Griseb.) W.R.Anderson & B.Gates | AF344531 | AF351073 | AJ403020 | AF500574 |
| *Blepharandra fimbriata* MacBryde |  | HQ246767 | HQ247447 | HQ246985 |
| *Blepharandra heteropetala* W.R.Anderson | AF344532 | AF351008 | AF344461 | AF500523 |
| *Brachylophon curtisii* Oliv. | HQ247215 | AF351018 | HQ247449 | AY137337 |
| *Bronwenia cornifolia* (Kunth) W.R. Anderson & C.Davis | HQ247217 | HQ246770 | HQ247451 | HQ246988 |
| *Bronwenia mathiasiae* (W.R.Anderson) W.R.Anderson & C.Davis | HQ247219 | HQ246772 | HQ247453 | HQ246990 |
| *Bunchosia glandulifera* (Jacq.) Kunth | HQ247225 | HQ246778 | HQ247454 | HQ246996 |
| *Bunchosia maritima* (Vell.) J.F.Macbr*.* | MG718606 |  | MG718053 |  |
| *Bunchosia montana* A.Juss*.* | KM197246 |  |  |  |
| *Bunchosia nitida* (Jacq.) A.Rich*.* |  | HQ246783 |  | HQ247002 |
| *Bunchosia polystachia* (Andrews) DC*.* | HQ247232 | HQ246786 |  | HQ247005 |
| *Burdachia sphaerocarpa* A.Juss*.* | AF344534 | AF351071 | AF344462 | AF500572 |
| *Byrsonima coccolobifolia* Kunth | HQ247237 | HQ246790 | HQ247460 | HQ247010 |
| *Byrsonima crassifolia* (L.) Kunth | AF344535 | AF351011 | L01892 | AF500526 |
| *Calcicola parvifolia* (A.Juss.) W.R.Anderson & C.Davis | HQ247243 | HQ246797 | HQ247465 | HQ247017 |
| *Callaeum antifebrile* (Ruiz ex Griseb.) D.M.Johnson | HQ247244 | HQ246798 | HQ247466 | HQ247018 |
| *Callaeum psilophyllum* (A.Juss.) D.M.Johnson | HQ247248 |  |  | HQ247023 |
| *Camarea axillaris* A.St.-Hil. | HQ247250 | AY499081 | HQ247471 | AY499055 |
| *Carolus chasei* (W.R.Anderson) W.R.Anderson | AF344564 | AF351054 | AF344496 | AF500558 |
| *Carolus sinemariensis* (Aubl.) W.R.Anderson | HQ247253 | HQ246806 | HQ247472 | HQ247027 |
| *Caucanthus auriculatus* (Radlk.) Nied. | HQ247254 | HQ246807 | HQ247473 | HQ247028 |
| *Christianella multiglandulosa* (Nied.) W.R.Anderson | HQ247255 | HQ246808 | HQ247474 | HQ247029 |
| *Coleostachys genipifolia* A.Juss. | AF344538 | AF351012 | AF344465 | AF500527 |
| *Cordobia argentea* (Griseb.) Nied*.* | AF344539 | AF351081 | AF344466 | HQ247031 |
| *Cottsia californica* (Benth.) W.R.Anderson & C.Davis | AF344554 | AF351031 | AF344486 | AF500539 |
| *Cottsia linearis* (Wiggins) W.R.Anderson & C.Davis | AF344555 | AF351030 | AF344487 | AF500538 |
| *Diacidia ferruginea* (Maguire & K.D.Phelps) W.R.Anderson | AF344540 | AF351010 | AF344467 | AF500525 |
| *Diaspis albida* Nied. *= Caucanthus albidus* (Nied.) Nied. | HQ247258 | HQ246812 | HQ247477 | HQ247033 |
| *Dicella macroptera* A.Juss. | HQ247262 | HQ246817 |  | HQ247038 |
| *Dicella nucifera* Chodat | AF344541 | AF351048 | AJ235802 | AF500553 |
| *Digoniopterys microphylla* Arènes | HQ247264 | HQ246818 | HQ247481 | HQ247040 |
| *Dinemagonum gayanum* A.Juss. | HQ247265 | AF351084 | AF344468 | HQ247041 |
| *Dinemandra ericoides* A.Juss. | AF344542 | AF351069 | AF344469 | AF500570 |
| *Diplopterys cabrerana* (Cuatrec.) B.Gates | HQ247266 | AF351039 | HQ247482 | AF500582 |
| *Diplopterys lutea* (Griseb.) W.R.Anderson & C.Davis | HQ247267 | HQ246819 | HQ247483 | HQ247043 |
| *Diplopterys pubipetala* (A.Juss.) W.R.Anderson & C.Davis | HQ247269 | HQ246821 | HQ247485 | HQ247045 |
| *Echinopterys eglandulosa (A.Juss.) Small* | AF344543 | AF351047 | AF344470 | AF500552 |
| *Echinopterys setosa* Brandegee | KM197264 | KM197367 |  |  |

**Supplementary table 1.** Continued

|  | Plastid | | | Nuclear |
| --- | --- | --- | --- | --- |
|  | *matK* | *ndhF* | *rbcL* | PHYC |
| *Ectopopterys soejartoi* W.R.Anderson | HQ247272 | AF351064 | AF344471 | AF500565 |
| *Excentradenia propinqua* (W.R.Anderson) W.R.Anderson |  | AF351062 |  |  |
| *Flabellaria paniculata* Cav. |  | AF351083 | AF344472 |  |
| *Flabellariopsis acuminata* (Engl.) R.Wilczek | HQ247274 | HQ246826 | AF344473 | HQ247050 |
| *Gallardoa fischeri* Hicken | AF344544 | AF351035 | AF344474 | AF500543 |
| *Galphimia glauca* Cav. |  |  |  | HQ247053 |
| *Galphimia gracilis* Bartl. | AF344545 | AF351015 | AF344475 | AF500530 |
| *Gaudichaudia albida* Schltdl. & Cham. | AF344546 | AF351034 | AF344476 | AF500542 |
| *Gaudichaudia mcvaughii* W.R.Anderson | AF344547 | AF351032 | AF344477 | AF500540 |
| *Glandonia macrocarpa* Griseb. | AF344548 | AF351072 | AF344478 | AF500573 |
| *Glicophyllum ambiguum* (A.Juss.) R.F.Almeida*= Tetrapterys ambigua* (A.Juss.) Nied*.* |  | HQ246926 | HQ247588 | HQ247155 |
| *Glicophyllum arcanum* (C.V.Morton) R.F.Almeida*= Tetrapterys arcana* C.V.Morton | HQ247396 | HQ246927 |  | HQ247156 |
| *Glicophyllum microphyllum* (Nied.) R.F.Almeida*= Tetrapterys microphylla* Nied. | AF344579 | AF351052 | AF344515 | AF500556 |
| *Heladena multiflora* (Hook. & Arn.) Nied. | AF344549 | AF351044 | AF344479 | AF500549 |
| *Henleophytum echinatum* (Griseb.) Small | HQ247281 | HQ246838 | HQ247492 |  |
| *Heteropterys bicolor* A.Juss. | HQ247284 | AY499084 | HQ247495 | AY499058 |
| *Heteropterys brachiata* (L.) DC. | HQ247285 | HQ246839 | HQ247496 | HQ247063 |
| *Heteropterys chrysophylla* (Lam.) Kunth | HQ247289 | AY499088 | HQ247500 | AY499062 |
| *Heteropterys cordifolia* Moric. | HQ247291 | HQ246841 | HQ247502 | HQ247065 |
| *Heteropterys glabra* Hook. & Arn. | HQ247293 | HQ246843 | HQ247504 | HQ247067 |
| *Heteropterys imperata* Amorim | HQ247294 | HQ246844 | HQ247505 | HQ247068 |
| *Heteropterys leona* (Cav.) Exell | HQ247295 | AF351050 | HQ247506 | AF500555 |
| *Heteropterys pteropetala* A.Juss*.* | HQ247303 | HQ246849 | HQ247514 | HQ247072 |
| *Heteropterys rhopalifolia* A.Juss. | HQ247305 | AY499093 | HQ247516 | AY499067 |
| *Heteropterys trichanthera* A.Juss. | HQ247311 | AY499099 | HQ247524 | AY499073 |
| *Hiptage benghalensis* (L.) Kurz | HQ247313 | AF351057 | HQ247525 | AF500561 |
| *Hiraea fagifolia* (DC.) A.Juss. | AF344552 | AF351060 | AF344483 | AF500564 |
| *Hiraea smilacina* Standl. |  | AF351061 | AF344484 | HQ247080 |
| *Janusia anisandra* (A. Juss.) Griseb. | AF344553 | AF351028 | AF344485 | AF500536 |
| *Janusia guaranitica* (A.St.-Hil.) A.Juss. | HQ247322 | HQ246861 | HQ247531 | HQ247085 |
| *Janusia janusioides* W.R. Anderson |  | HQ246863 | HQ247533 | HQ247087 |
| *Janusia linearifolia* (A. St.-Hil.) A. Juss*.* = *Peregrina linearifolia* (A.St.-Hil.) W.R.Anderson | AF344572 | AY499102 | AF344505 | AF500535 |
| *Janusia mediterranea* (Vell.) W.R.Anderson | AF344556 | AF351029 | AF344488 | AF500537 |
| *Jubelina rosea* (Miq.) Nied. | AF344557 | AF351079 | AF344489 | HQ247090 |
| *Jubelina wilburii* W.R. Anderson | HQ247327 | AY499100 | HQ247536 | AY499074 |
| *Lasiocarpus* sp. | HQ247328 | HQ246867 | HQ247537 | HQ247092 |
| *Lasiocarpus* sp. | AF344558 | AF351066 | AF344490 | AF500567 |
| *Lophanthera hammelii* W.R. Anderson | HQ247329 | HQ246868 | HQ247538 |  |
| *Lophanthera lactescens* Ducke | AF344559 | AF351009 | AF344491 | AF500524 |
| *Lophanthera longifolia* (Kunth) Griseb. | HQ247330 | HQ246869 | HQ247539 | HQ247094 |
| *Lophopterys floribunda* W.R. Anderson & C. Davis | AF344560 | AF351078 | AF344492 | AF500579 |
| *Madagasikaria andersonii* C. Davis | HQ247333 | AF436790 | HQ247541 | AF436800 |
| *Malpighia albiflora* (Cuatrec.) Cuatrec. | HQ247334 | HQ246872 | HQ247542 | HQ247097 |
| *Malpighia emarginata* DC. | AF344561 | AF351023 | AF344493 | AF436799 |
| *Malpighia glabra* L. | HQ247337 | HQ246876 | HQ247544 | HQ247101 |
| *Malpighia incana* Mill. | HQ247338 | HQ246877 | HQ247545 | HQ247102 |
| *Malpighia leticiana* (W.R.Anderson) W.R.Anderson & C.Davis | HQ247339 | HQ246878 | HQ247546 | HQ247103 |
| *Malpighia mexicana* A. Juss. | HQ247340 | HQ246879 |  | HQ247104 |
| *Malpighia stevensii* W.R. Anderson | HQ247344 | AF351022 | HQ247547 | AF436798 |
| *Malpighiodes bracteosa* (Griseb.) W.R. Anderson | AF344563 | AF351055 | AF344495 | AF500559 |
| *Mascagnia arenicola* C.E. Anderson | HQ247347 | HQ246885 | HQ247548 | HQ247110 |
| *Mascagnia australis* C.E. Anderson | HQ247348 | HQ246886 | HQ247549 | HQ247111 |
| *Mascagnia brevifolia* Griseb*.* | HQ247349 | HQ246887 | HQ247550 | HQ247112 |
| *Mascagnia cordifolia* (A. Juss.) Griseb*.* | HQ247350 | HQ246888 | HQ247351 | HQ247113 |
| *Mascagnia divaricata* (Kunth) Nied*.* | HQ247352 | HQ246890 | HQ247553 | HQ247115 |
| *Mascagnia polybotrya* Nied*.* | HQ247355 | HQ246893 | HQ247556 | HQ247118 |
| *Mascagnia vacciniifolia* Nied*.* | HQ247359 | HQ246897 | HQ247558 | HQ247122 |
| *Mcvaughia bahiana* W.R. Anderson | AF344568 | AF351070 | AF344501 | AF500571 |
| *Mezia araujoi* Schwacke ex Nied*.* | AF344569 | AF351051 | AF344502 | HQ247123 |
| *Mezia includens* (Benth.) Cuatrec. | HQ247360 | HQ246898 | HQ247559 | HQ247124 |

**Table S1.** Continued

|  | Plastid | | | Nuclear |
| --- | --- | --- | --- | --- |
|  | *matK* | *ndhF* | *rbcL* | PHYC |
| *Microsteira ambovombensis* Arènes | HQ247361 | HQ246899 | HQ247561 | HQ247125 |
| *Mionandra camareoides* Griseb. | AF344570 | AF351080 | AF344503 | AF500580 |
| *Niedenzuella acutifolia* (Cav.) W.R.Anderson | HQ247365 | HQ246903 | HQ247563 | HQ247129 |
| *Niedenzuella sericea* (A. Juss.) W.R. Anderson | HQ247367 | HQ246905 | HQ247565 | HQ247131 |
| *Niedenzuella stannea* (Griseb.) W.R.Anderson | HQ247369 | HQ246906 | HQ247566 | HQ247132 |
| *Peixotoa cordistipula* A. Juss. | HQ247370 | HQ246907 | HQ247567 | HQ247133 |
| *Peixotoa glabra* A. Juss*.* | AF344571 | AF351036 | AF344504 | AF500544 |
| *Philgamia glabrifolia* Arènes | HQ247374 | HQ246910 | HQ247568 | HQ247137 |
| *Psychopterys dipholiphylla* (Small) W.R. Anderson & S. Corso | AF344565 | AF351063 | AF344497 | AF436795 |
| *Pterandra arborea* Ducke | AF344573 | AF351014 | AF344506 | AF500529 |
| *Ptilochaeta bahiensis* Turcz. | AF344574 | AF351068 | AF344507 | AF500569 |
| *Ptilochaeta nudipes* Griseb. | HQ247376 | AF351067 | HQ247570 | AF500568 |
| *Rhynchophora humbertii* Arènes | HQ247377 | HQ246912 | HQ247571 | HQ247139 |
| *Rhynchophora phillipsonii* W.R. Anderson | HQ247378 | AF436793 | HQ247572 | AF436802 |
| *Ryssopterys intermedia* Hochr. = *Ryssopterys timoriensis* (DC.) Blume ex A. Juss. |  | AF351040 | AF344509 | AF500545 |
| *Ryssopterys tiliifolia* Blume ex A.Juss. |  | HQ246914 | HQ247574 | HQ247141 |
| *Spachea correae* Cuatrec. & Croat | AF344575 | AF351074 | AF344510 | AF500575 |
| *Spachea elegans* (G. Mey.) A. Juss*.* | HQ247380 | HQ246915 | HQ247575 | HQ247142 |
| *Sphedamnocarpus pruriens* (A. Juss.) Szyszył*.* | HQ247383 |  | HQ247578 | HQ247146 |
| *Stigmaphyllon calcaratum* N.E. Br*.* | HQ247389 | HQ246922 | HQ247583 | HQ247150 |
| *Stigmaphyllon ciliatum* (Lam.) A.Juss*.* | HQ247390 | HQ246923 | HQ247584 | HQ247151 |
| *Stigmaphyllon lindenianum* A.Juss. | HQ247392 |  | HQ247586 | HQ247153 |
| *Tetrapterys discolor* (G.Mey.) DC*.* | AF344578 | AF351076 |  | AF500577 |
| *Tetrapterys phlomoides* (Spreng.) Nied. | HQ247399 | HQ246930 | HQ247591 | HQ247159 |
| *Tetrapterys schiedeana* Schltdl. & Cham*.* | HQ247400 | HQ246932 | HQ247593 | HQ247161 |
| *Tetrapterys tinifolia* Triana & Planch*.* | HQ247401 | HQ246933 | HQ247594 | HQ247162 |
| *Thryallis longifolia* Mart. | AF344580 | AF351046 | AF344516 | AF500551 |
| *Triaspis hypericoides* Burch. | HQ247404 | HQ246936 | HQ247596 | HQ247165 |
| *Tricomaria usillo* Hook. & Arn. | AF344582 | AF351075 | AF344518 | AF500576 |
| *Triopterys jamaicensis* L. | AF344583 | AF351024 | AF344519 | AF500534 |
| *Triopterys paniculata* Small | HQ247407 | HQ246940 | HQ247599 | HQ247169 |
| *Tristellateia africana* S. Moore | HQ247408 | AF351043 | HQ247600 | AF500548 |
| *Tristellateia australasiae* A. Rich. | MF350152 |  | MF349685 |  |
| *Tristellateia madagascariensis* Poir*.* | AF344584 | AF351042 | AF344520 | AF500547 |
| *Verrucularia glaucophylla* A. Juss. | HQ247414 | AY499108 | HQ247602 | AY499080 |
| **Centroplacaceae** |  |  |  |  |
| *Centroplacus glaucinus* Pierre | FJ670002 | FJ670066 | AY663646 | FJ669884 |
| **Chrysobalanaceae** |  |  |  |  |
| *Atuna racemosa* Raf. | EF135503 | AY425030 | AF089758 | AY425088 |
| **Elatinaceae** |  |  |  |  |
| *Bergia pedicellaris* (F.Muell.) F.Muell. ex Benth. | HQ247213 | HQ246766 | HQ247446 | HQ246984 |
| *Elatine triandra* Schkuhr | EF135532 | AY425049 | AY380348 | AY425104 |
| **Euphorbiaceae** |  |  |  |  |
| *Acalypha californica* Benth. | EF135499 | AY425027 | AY380341 | AY425087 |
| **Goupiaceae** |  |  |  |  |
| *Goupia glabra* Aubl. | EF135544 | AY425054 | AJ235780 | AY425108 |
| **Ochnaceae** |  |  |  |  |
| *Ochna* sp | FJ670031 | AY425073 | AY380354 | AY425123 |
| **Phyllanthaceae** |  |  |  |  |
| *Phyllanthus calycinus* Labill. | EF135580 | EU002250 | AY663603 | AY579869 |
| **Picrodendraceae** |  |  |  |  |
| *Androstachys johnsonii* Prain |  | AF500495 | AJ402922 | AF500522 |
| **Putranjivaceae** |  |  |  |  |
| *Putranjiva roxburghii* Wall. | EF135530 | AY425048 | M95757 | AY425102 |
| **Violaceae** |  |  |  |  |
| *Hymenanthera alpina* (Kirk) W.R.B.Oliv. | EF135552 | AY425059 | Z75692 | AY425112 |
| **Celastrales** |  |  |  |  |
| **Celastraceae** |  |  |  |  |
| *Denhamia celastroides* (F.Muell.) Jessup | EF135526 | AY425043 | AJ402941 | AY425097 |
| **Saxifragales** |  |  |  |  |
| **Peridiscaceae** |  |  |  |  |
| *Peridiscus lucidus* Benth. | DQ411570 | AY425076 | AY380356 | AY425125 |
