# Supplementary material for: Phloem wedges in Malpighiaceae: origin, structure, diversification, and systematic relevance
Source: EvoDevo. 2022 Apr 28;13:11. doi: 10.1186/s13227-022-00196-3 (PMC9052467; doi:10.1186/s13227-022-00196-3)

**Additional file 2**

**Article title**: Phloem wedges in Malpighiaceae: origin, structure, diversification, and systematic relevance

**Authors:** Quintanar-Castillo A and Pace MR, 2022

**Figure. S1.** Maximum clade credibility tree (MCC) derived from BEAST analyses with divergence time estimates for Malpighiaceae. Numbers are the mean estimated ages of clades (Ma). The purple bars on the tree represent the 95% highest posterior density (HPD) indexes.


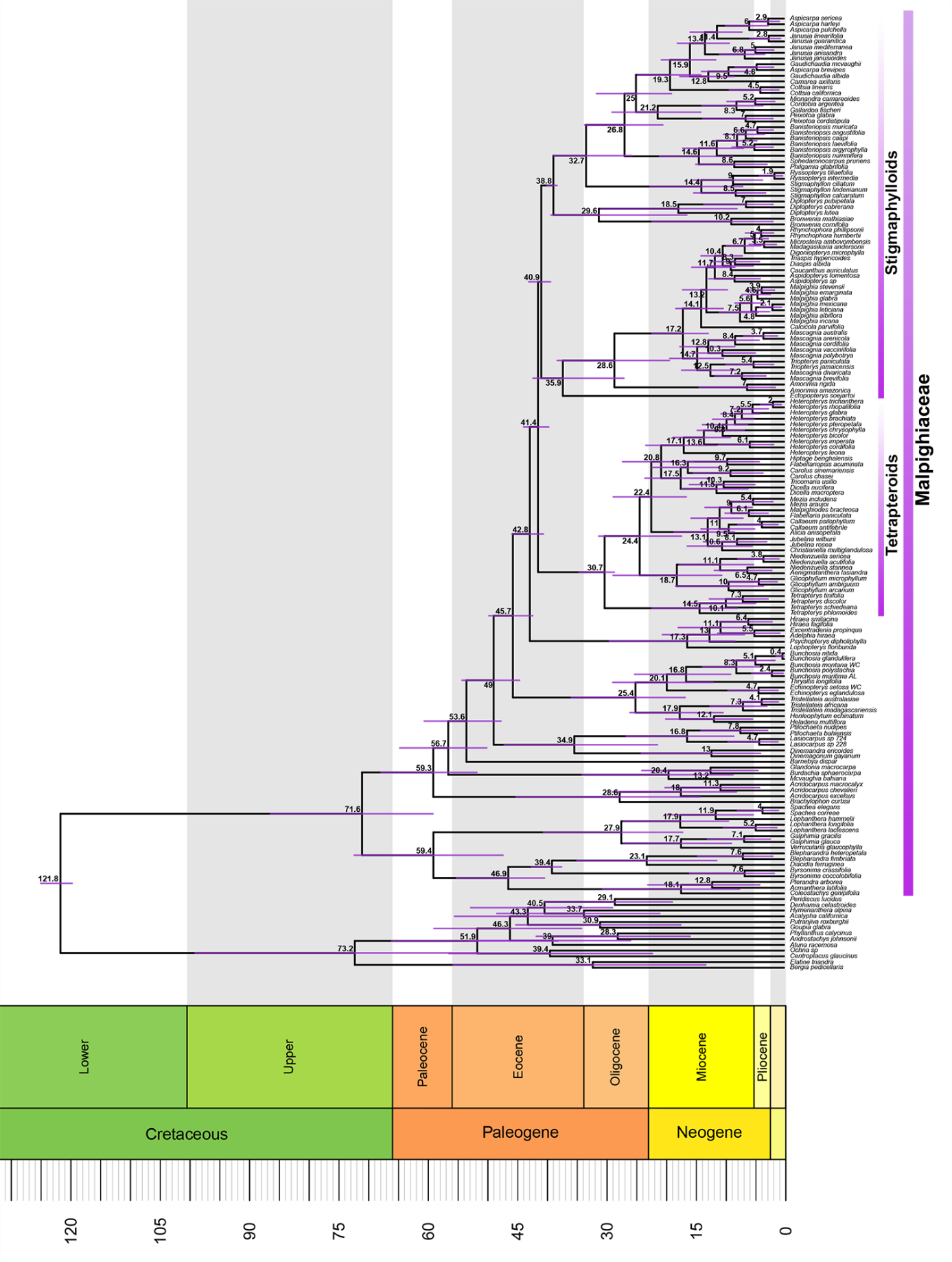

Supplement: Supplementary file 2 — Additional file 2: Figure S1. Maximum clade credibility tree (MCC) with divergence time estimates for Malpighiaceae. [file 13227_2022_196_MOESM2_ESM.docx]
