# Supplementary material for: Phloem wedges in Malpighiaceae: origin, structure, diversification, and systematic relevance
Source: EvoDevo. 2022 Apr 28;13:11. doi: 10.1186/s13227-022-00196-3 (PMC9052467; doi:10.1186/s13227-022-00196-3)
Supplement: Supplementary file 3 — Additional file 3: Table S3.1. Character data set used for the ancestral state reconstruction [file 13227_2022_196_MOESM3_ESM.docx]

# **Additional file 3**

**Article title**: Phloem wedges in Malpighiaceae: origin, structure, diversification, and systematic relevance

**Authors:** Quintanar-Castillo A and Pace MR, 2022

**Table S3.1.** Habit and phloem wedges in the stem of the sampled taxa in Malpighiaceae. The information was compiled from herbarium records, literature, and our personal observations.

| **Species** | **Habit** | **Phloem wedges in stem** |
| --- | --- | --- |
| *Acalypha californica* | Self-supporting | Absent |
| *Acmanthera latifolia* | Self-supporting | Absent |
| *Acridocarpus chevalieri* | Climber | Absent |
| *Acridocarpus excelsus* | Self-supporting | Absent |
| *Acridocarpus macrocalyx* | Climber | Absent |
| *Adelphia hiraea* | Climber | Absent |
| *Aenigmatanthera lasiandra* | Climber | Absent |
| *Alicia anisopetala* | Climber | Present |
| *Amorimia amazonica* | Climber | Absent |
| *Amorimia rigida* | Climber | Absent |
| *Androstachys johnsonii* | Self-supporting | Absent |
| *Aspicarpa brevipes* | Self-supporting | Absent |
| *Aspicarpa harleyi* | Self-supporting | Absent |
| *Aspicarpa pulchella* | Self-supporting | Absent |
| *Aspicarpa sericea* | Self-supporting | Absent |
| *Aspidopterys sp* | Climber | Absent |
| *Aspidopterys tomentosa* | Climber | Absent |
| *Atuna racemosa* | Self-supporting | Absent |
| *Banisteriopsis angustifolia* | Self-supporting | Absent |
| *Banisteriopsis argyrophylla* | Climber | Present |
| *Banisteriopsis caapi* | Climber | Present |
| *Banisteriopsis laevifolia* | Climber | Absent |
| *Banisteriopsis muricata* | Climber | Absent |
| *Banisteriopsis nummifera* | Climber | Absent |
| *Barnebya dispar* | Self-supporting | Absent |
| *Bergia pedicellaris* | Self-supporting | Absent |
| *Blepharandra fimbriata* | Self-supporting | Absent |
| *Blepharandra heteropetala* | Self-supporting | Absent |
| *Brachylophon curtisii* | Self-supporting | Absent |
| *Bronwenia cornifolia* | Climber | Absent |
| *Bronwenia mathiasiae* | Climber | Absent |
| *Bunchosia glandulifera* | Self-supporting | Absent |

**Table S3.1.** Continued

| **Species** | **Habit** | **Phloem wedges in stem** |
| --- | --- | --- |
| *Bunchosia maritima* | Self-supporting | Absent |
| *Bunchosia montana* | Self-supporting | Absent |
| *Bunchosia nitida* | Self-supporting | Absent |
| *Bunchosia polystachia* | Self-supporting | Absent |
| *Burdachia sphaerocarpa* | Self-supporting | Absent |
| *Byrsonima coccolobifolia* | Self-supporting | Absent |
| *Byrsonima crassifolia* | Self-supporting | Absent |
| *Calcicola parvifolia* | Self-supporting | Absent |
| *Callaeum antifebrile* | Climber | Present |
| *Callaeum psilophyllum* | Climber | Present |
| *Camarea axillaris* | Self-supporting | Absent |
| *Carolus chasei* | Climber | Present |
| *Carolus sinemariensis* | Climber | Present |
| *Caucanthus auriculatus* | Climber | Absent |
| *Centroplacus glaucinus* | Self-supporting | Absent |
| *Christianella multiglandulosa* | Climber | Present |
| *Coleostachys genipifolia* | Self-supporting | Absent |
| *Cordobia argentea* | Climber | Absent |
| *Cottsia californica* | Climber | Absent |
| *Cottsia linearis* | Climber | Absent |
| *Denhamia celastroides* | Self-supporting | Absent |
| *Diacidia ferruginea* | Self-supporting | Absent |
| *Diaspis albida* | Climber | Absent |
| *Dicella macroptera* | Climber | Absent |
| *Dicella nucifera* | Climber | Absent |
| *Digoniopterys microphylla* | Self-supporting | Absent |
| *Dinemagonum gayanum* | Self-supporting | Absent |
| *Dinemandra ericoides* | Self-supporting | Absent |
| *Diplopterys cabrerana* | Climber | Absent |
| *Diplopterys lutea* | Climber | Absent |
| *Diplopterys pubipetala* | Climber | Present |
| *Echinopterys eglandulosa* | Climber | Absent |
| *Echinopterys setosa* | Self-supporting | Absent |
| *Ectopopterys soejartoi* | Climber | Absent |
| *Elatine triandra* | Self-supporting | Absent |
| *Excentradenia propinqua* | Climber | Absent |
| *Flabellaria paniculata* | Climber | Present |
| *Flabellariopsis acuminata* | Climber | Absent |
| *Gallardoa fischeri* | Self-supporting | Absent |
| *Galphimia glauca* | Self-supporting | Absent |
| *Galphimia gracilis* | Self-supporting | Absent |
| *Gaudichaudia albida* | Climber | Absent |
| *Gaudichaudia mcvaughii* | Climber | Absent |

**Table S3.1.** Continued

| **Species** | **Habit** | **Phloem wedges in stem** |
| --- | --- | --- |
| *Glandonia macrocarpa* | Self-supporting | Absent |
| *Glicophyllum ambiguum* | Climber | Absent |
| *Glicophyllum arcanum* | Climber | Absent |
| *Glicophyllum microphyllum* | Climber | Absent |
| *Goupia glabra* | Self-supporting | Absent |
| *Heladena multiflora* | Climber | Absent |
| *Henleophytum echinatum* | Climber | Absent |
| *Heteropterys bicolor* | Climber | Absent |
| *Heteropterys brachiata* | Climber | Absent |
| *Heteropterys chrysophylla* | Climber | Absent |
| *Heteropterys cordifolia* | Climber | Present |
| *Heteropterys glabra* | Self-supporting | Absent |
| *Heteropterys imperata* | Climber | Absent |
| *Heteropterys leona* | Climber | Absent |
| *Heteropterys pteropetala* | Self-supporting | Absent |
| *Heteropterys rhopalifolia* | Climber | Present |
| *Heteropterys trichanthera* | Climber | Absent |
| *Hiptage benghalensis* | Climber | Absent |
| *Hiraea fagifolia* | Climber | Present |
| *Hiraea smilacina* | Climber | Absent |
| *Hymenanthera alpina* | Self-supporting | Absent |
| *Janusia anisandra* | Climber | Absent |
| *Janusia guaranitica* | Climber | Present |
| *Janusia janusioides* | Climber | Absent |
| *Janusia linearifolia* | Climber | Absent |
| *Janusia mediterranea* | Climber | Absent |
| *Jubelina rosea* | Climber | Present |
| *Jubelina wilburii* | Climber | Present |
| *Lasiocarpus* sp 228 | Self-supporting | Absent |
| *Lasiocarpus* sp 724 | Self-supporting | Absent |
| *Lophanthera hammelii* | Self-supporting | Absent |
| *Lophanthera lactescens* | Self-supporting | Absent |
| *Lophanthera longifolia* | Self-supporting | Absent |
| *Lophopterys floribunda* | Climber | Absent |
| *Madagasikaria andersonii* | Climber | Absent |
| *Malpighia albiflora* | Self-supporting | Absent |
| *Malpighia emarginata* | Self-supporting | Absent |
| *Malpighia glabra* | Self-supporting | Absent |
| *Malpighia incana* | Self-supporting | Absent |
| *Malpighia leticiana* | Self-supporting | Absent |
| *Malpighia mexicana* | Self-supporting | Absent |
| *Malpighia stevensii* | Self-supporting | Absent |
| *Malpighiodes bracteosa* | Climber | Present |

**Table S3.1.** Continued

| **Species** | **Habit** | **Phloem wedges in stem** |
| --- | --- | --- |
| *Mascagnia arenicola* | Climber | Present |
| *Mascagnia australis* | Climber | Present |
| *Mascagnia brevifolia* | Climber | Present |
| *Mascagnia cordifolia* | Climber | Present |
| *Mascagnia divaricata* | Climber | Absent |
| *Mascagnia polybotrya* | Climber | Present |
| *Mascagnia vacciniifolia* | Climber | Present |
| *Mcvaughia bahiana* | Self-supporting | Absent |
| *Mezia araujoi* | Climber | Present |
| *Mezia includens* | Climber | Present |
| *Microsteira ambovombensis* | Self-supporting | Absent |
| *Mionandra camareoides* | Self-supporting | Absent |
| *Niedenzuella acutifolia* | Climber | Present |
| *Niedenzuella sericea* | Climber | Present |
| *Niedenzuella stannea* | Climber | Present |
| *Ochna sp* | Self-supporting | Absent |
| *Peixotoa cordistipula* | Climber | Absent |
| *Peixotoa glabra* | Climber | Present |
| *Peridiscus lucidus* | Self-supporting | Absent |
| *Philgamia glabrifolia* | Climber | Absent |
| *Phyllanthus calycinus* | Self-supporting | Absent |
| *Psychopterys dipholiphylla* | Climber | Absent |
| *Pterandra arborea* | Self-supporting | Absent |
| *Ptilochaeta bahiensis* | Self-supporting | Absent |
| *Ptilochaeta nudipes* | Self-supporting | Absent |
| *Putranjiva roxburghii* | Self-supporting | Absent |
| *Rhynchophora humbertii* | Climber | Absent |
| *Rhynchophora phillipsonii* | Climber | Absent |
| *Ryssopterys intermedia* | Climber | Absent |
| *Ryssopterys tiliaefolia* | Climber | Absent |
| *Spachea correae* | Self-supporting | Absent |
| *Spachea elegans* | Self-supporting | Absent |
| *Sphedamnocarpus pruriens* | Climber | Absent |
| *Stigmaphyllon calcaratum* | Climber | Absent |
| *Stigmaphyllon ciliatum* | Climber | Absent |
| *Stigmaphyllon lindenianum* | Climber | Present |
| *Tetrapterys discolor* | Climber | Absent |
| *Tetrapterys phlomoides* | Climber | Absent |
| *Tetrapterys schiedeana* | Climber | Absent |
| *Tetrapterys tinifolia* | Climber | Absent |
| *Thryallis longifolia* | Climber | Absent |
| *Triaspis hypericoides* | Climber | Absent |
| *Tricomaria usillo* | Self-supporting | Absent |

**Table S3.1.** Continued

| **Species** | **Habit** | **Phloem wedges in stem** |
| --- | --- | --- |
| *Triopterys jamaicensis* | Climber | Absent |
| *Triopterys paniculata* | Climber | Absent |
| *Tristellateia africana* | Climber | Present |
| *Tristellateia australasiae* | Climber | Present |
| *Tristellateia madagascariensis* | Climber | Present |
| *Verrucularia glaucophylla* | Self-supporting | Absent |

## **Growth form**

**Climber:** Plants that do not stand upright by themselves in their adult stages, and that climb different structures which use as support to grow in height, maintaining the connection with the ground temporarily or permanently [1-3]

**Self-Supporting**: Plants with autonomous mechanical support such as trees and shrubs with rigid and erect stems [1,4]

**References**

1. Cabanillas PA, Hurrel JA. Plantas trepadoras: tipo biológico y clasificación. Ciencias morfólogicas 2012; 14(2): 1-15
2. Beentje H. The Kew Plant Glossary: an illustrated dictionary of plant terms. Kew Publishing, Royal Botanic Garden, London. 2010.
3. Sperotto P, Acevedo-Rodríguez P, Vasconcelos TN, Roque N. Towards a standardization of terminology of the climbing habit in plants. Bot. Rev. The 2020; 86(3), 180-210.
4. Rowe N., Speck T. Plant growth forms: an ecological and evolutionary perspective. New Phytol. 2005 166: 61-72.

**Table S3.2.** Herbarium specimens revisited

| **Species** | **Collector** | **Collector number** | **Year** | **Country** | **Herbarium code** |
| --- | --- | --- | --- | --- | --- |
| *Acmanthera latifolia* (A.Juss.) Griseb. | Lohmann, LG | 109 | 1998 | Brazil | SPF |
| *Acmanthera latifolia* (A.Juss.) Griseb. | Fróes, RL | 29424 | 1953 | Brazil | IAN |
| *Acmanthera latifolia* (A.Juss.) Griseb. | Pires, JM | 13884 | 1974 | Brazil | IAN |
| *Acmanthera latifolia* (A.Juss.) Griseb. | Oliveira, AA | 2723 | 1995 | Brazil | SPF |
| *Acmanthera latifolia* (A.Juss.) Griseb. | Kuhlmann, JG | 1017 | 1923 | Brazil | RB |
| *Acridocarpus chevalieri* Sprague | Chevalier, AJB | 3144 | 1899 | Mali | P |
| *Acridocarpus chevalieri* Sprague | Duvall, CS | 249 | 1999 | Mali | MICH |
| *Acridocarpus chevalieri* Sprague | Duvall, CS | 121 | 1999 | Mali | MICH |
| *Acridocarpus chevalieri* Sprague | Birnbaum, P | 1228 | 2007 | Mali | P |
| *Acridocarpus chevalieri* Sprague | Birnbaum, P | 700 | 2003 | Mali | P |
| *Acridocarpus excelsus* A.Juss. | Humbert, H | 11708 | 1933 | Madagascar | P |
| *Acridocarpus excelsus* A.Juss. | Letsara, R | 888 | 2009 | Madagascar | P |
| *Acridocarpus excelsus* A.Juss. | Decary, R | 9314 | 1931 | Madagascar | P |
| *Acridocarpus excelsus* A.Juss. | Du Puy, B | 760 | 1990 | Madagascar | P |
| *Acridocarpus excelsus* A.Juss. | Phillipson, PB | 2255 | 1987 | Madagascar | P |
| *Acridocarpus macrocalyx* Engl. | Zenker, GA | 2472 | 1902 | Cameroon | P |
| *Acridocarpus macrocalyx* Engl | Le Testu, GM | 4680 | 1923 | Central African Republic | P |
| *Acridocarpus macrocalyx* Engl | Klaine, TJ | 2049 | 1900 | Gabon | P |
| *Acridocarpus macrocalyx* Engl | Letouzey, R | 11775 | 1973 | Cameroon | P |
| *Acridocarpus macrocalyx* Engl | Satabié, B | 748 | 1984 | Cameroon | P |
| *Adelphia hiraea* (Gaertn.) W.R.Anderson | Caxambu, MG | 5409 | 2014 | Brazil | HCF |
| *Adelphia hiraea* (Gaertn.) W.R.Anderson | Levy, S & Durán, A | 180 | 1994 | Mexico | MEXU |
| *Adelphia hiraea* (Gaertn.) W.R.Anderson | Ventura, F | 21026 | 1984 | Mexico | MEXU |
| *Adelphia hiraea* (Gaertn.) W.R.Anderson | Zamudio, S | 872 | 1983 | Mexico | MEXU |
| *Adelphia hiraea* (Gaertn.) W.R.Anderson | Martínez, E | 12194 | 1985 | Mexico | MEXU |
| *Amorimia amazonica* (Nied.) W.R.Anderson | Acevedo-Rodríguez, P | 14780 | 2009 | Brazil | RB |
| *Amorimia amazonica* (Nied.) W.R.Anderson | Costa, DS | 271 | 2013 | Brazil | RB |
| *Amorimia amazonica* (Nied.) W.R.Anderson | Daly, DC | 13263 | 2008 | Brazil | RB |
| *Amorimia amazonica* (Nied.) W.R.Anderson | Medeiros, H | 2131 | 2017 | Brazil | RB |
| *Amorimia amazonica* (Nied.) W.R.Anderson | Daly, DC | 9637 | 1997 | Brazil | NY |
| *Aspicarpa pulchella* (Griseb.) O'Donell & Lourteig | Souza, JP | 622 | 1996 | Brazil | ESA |
| *Aspicarpa pulchella* (Griseb.) O'Donell & Lourteig | Hatschbach, G | 35203 | 1974 | Brazil | MICH |
| *Aspicarpa pulchella* (Griseb.) O'Donell & Lourteig | Hatschbach, G | 39213 | 1976 | Brazil | MICH |
| *Aspicarpa pulchella* (Griseb.) O'Donell & Lourteig | Kummrow, R & Anderson WR | 1098 | 1976 | Brazil | MICH |
| *Aspicarpa pulchella* (Griseb.) O'Donell & Lourteig | Kummrow, R & Stutts, JG | 1779 | 1982 | Brazil | MICH |
| *Aspicarpa sericea* Griseb. | Hatschbach, G | 74228 | 2002 | Brazil | SPF |
| *Aspicarpa sericea* Griseb. | Scaramuzza, CAM | 191 | 1989 | Brazil | ESA |

**Table S3.2.** Continued

| **Species** | **Collector** | **Collector number** | **Year** | **Country** | **Herbarium code** |
| --- | --- | --- | --- | --- | --- |
| *Aspicarpa sericea* Griseb. | Souza, VC | 3550 | 1993 | Brazil | ESA |
| *Aspicarpa sericea* Griseb. | Nee, M & Mendoza JM | 51865 | 2001 | Bolivia | MICH |
| *Aspicarpa sericea* Griseb. | Elias, SI | 204 | 1997 | Brazil | ESA |
| *Aspidopterys tomentosa* (Blume) A.Juss. | Damoeli, Koealoe & Toroes | 1507 | 1928 | Indonesia | MICH |
| *Aspidopterys tomentosa* (Blume) A.Juss. | Soejarto, DD | 10597 | 1999 | Vietnam | P |
| *Aspidopterys tomentosa* (Blume) A.Juss. | Soejarto, DD | 14008 | 2008 | Vietnam |  |
| *Aspidopterys tomentosa* (Blume) A.Juss. | Dubost, JM | 98 | 2010 | Laos | P |
| *Aspidopterys tomentosa* (Blume) A.Juss. | Dubost, JM | 75 | 2010 | Laos | P |
| *Banisteriopsis angustifolia* (A.Juss.) B.Gates | de Carvalho, AMV | 3709 | 1992 | Brazil | MBML |
| *Banisteriopsis angustifolia* (A.Juss.) B.Gates | Roque, N | 4076 | 2014 | Brazil | US |
| *Banisteriopsis angustifolia* (A.Juss.) B.Gates | Leitão Filho, HF | 27686 | 1992 | Brazil | HUFU |
| *Banisteriopsis angustifolia* (A.Juss.) B.Gates | Amorim, AM | 2778 | 1999 | Brazil | MBML |
| *Banisteriopsis argyrophylla* (A.Juss.) B.Gates | Alves Rezende, A | 458 | 1996 | Brazil | SJRP |
| *Banisteriopsis argyrophylla* (A.Juss.) B.Gates | Cordeiro, J | 4907 | 2013 | Brazil | HUEFS |
| *Banisteriopsis argyrophylla* (A.Juss.) B.Gates | Pace, MR | 2013 | 2013 | Brazil | US |
| *Banisteriopsis argyrophylla* (A.Juss.) B.Gates | Dawson, EY | 14198 | 1956 | Brazil | P |
| *Banisteriopsis argyrophylla* (A.Juss.) B.Gates | Santos, RR | 1243 | 1968 | Brazil | P |
| *Barnebya dispar* (Griseb.) W.R.Anderson & B.Gates | Silva Neto, SJ | 1674 | 2002 | Brazil | RB |
| *Barnebya dispar* (Griseb.) W.R.Anderson & B.Gates | Kurtz, BC | 253 | 1993 | Brazil | RB |
| *Barnebya dispar* (Griseb.) W.R.Anderson & B.Gates | Bovini, MG | 2168 | 2002 | Brazil | RB |
| *Barnebya dispar* (Griseb.) W.R.Anderson & B.Gates | Silva Neto, SJ | 1539 | 2001 | Brazil | RB |
| *Barnebya dispar* (Griseb.) W.R.Anderson & B.Gates | Leoni, LS | 4156 | 1999 | Brazil | RB |
| *Carolus sinemariensis* (Aubl.) W.R.Anderson | Rabelo, BV | 1820 | 1983 | Brazil | NY |
| *Carolus sinemariensis* (Aubl.) W.R.Anderson | Killip, EP | 30643 | 1929 | Brazil | NY |
| *Carolus sinemariensis* (Aubl.) W.R.Anderson | Herrera, G | 636 | 1987 | Mexico | MEXU |
| *Carolus sinemariensis* (Aubl.) W.R.Anderson | Martínez, E | 19012 | 1986 | Mexico | MEXU |
| *Carolus sinemariensis* (Aubl.) W.R.Anderson | Álvarez, D | 5974 | 2003 | Mexico | MEXU |
| *Caucanthus auriculatus* (Radlk.) Nied. | Knox, E | 2128 | 1991 | Kenya | MICH |
| *Centroplacus glaucinus* Pierre | de Wilde, JJ | 8251 | 1975 | Cameroon | MBM |
| *Flabellaria paniculata* Cav. | Zenker, GA |  | 1909 | Cameroon | MICH |
| *Gallardoa fischeri* Hicken | Anderson, WR | 13580 | 1990 | Argentina | MICH |
| *Heteropterys chrysophylla* (Lam.) Kunth | Machado, DNS | 352 | 2013 | Brazil | RB |
| *Heteropterys chrysophylla* (Lam.) Kunth | Feteira, PW | 240 | 2004 | Brazil | RB |

**Table S3.2.** Continued

| **Species** | **Collector** | **Collector number** | **Year** | **Country** | **Herbarium code** |
| --- | --- | --- | --- | --- | --- |
| *Heteropterys chrysophylla* (Lam.) Kunth | Amorim, AM | 39 | 1988 | Brazil | RB |
| *Heteropterys chrysophylla* (Lam.) Kunth | Amorim, AM | 73 | 1989 | Brazil | RB |
| *Heteropterys chrysophylla* (Lam.) Kunth | Farney, c | 2217 | 1988 | Brazil | RB |
| *Heteropterys cordifolia* Moric. ex A.Juss. | Amorim, AM | 3095 | 1999 | Brazil | RB |
| *Heteropterys cordifolia* Moric. ex A.Juss. | Farias, GL | 185 | 1988 | Brazil | RB |
| *Heteropterys cordifolia* Moric. ex A.Juss. | Amorim, AM | 3087 | 1999 | Brazil | NY |
| *Heteropterys cordifolia* Moric. ex A.Juss. | Amorim, AM | 3417 | 2000 | Brazil | NY |
| *Heteropterys cordifolia* Moric. ex A.Juss. | de Carvalho, AMV | 3393 | 1991 | Brazil | NY |
| *Heteropterys leona* (Cav.) Exell | Almeida, TE | 4068 | 2015 | Brazil | HSTM |
| *Heteropterys leona* (Cav.) Exell | Silveira, ALP | 466 | 2013 | Brazil | RB |
| *Heteropterys leona* (Cav.) Exell | Andre, T | 80 | 2015 | Brazil | HSTM |
| *Heteropterys leona* (Cav.) Exell | Giacomin, LL | 2545 | 2015 | Brazil | HSTM |
| *Heteropterys leona* (Cav.) Exell | Santos, LO | 453 | 2008 | Brazil | IAN |
| *Heteropterys leona* (Cav.) Exell | Giacomin, LL | 2545 | 2015 | Brazil | RB |
| *Heteropterys leona* (Cav.) Exell | Forzza, RC | 6179 | 2011 | Brazil | RB |
| *Hiraea fagifolia* (DC.) A.Juss. | Allen, B | 15184 | 1993 | Belize | MO |
| *Hiraea fagifolia* (DC.) A.Juss. | Sinaca Colín, S | 787 | 1986 | Mexico | MEXU |
| *Hiraea fagifolia* (DC.) A.Juss. | Zamudio, S & Guadarrama, MA | 860 | 1983 | Mexico | MEXU |
| *Hiraea fagifolia* (DC.) A.Juss. | Gentle, PH | 2760 | 1939 | Belize | MEXU |
| *Hiraea fagifolia* (DC.) A.Juss. | Wendt, TL | 3660 | 1982 | Mexico | MEXU |
| *Hiraea smilacina* Standl. | Hammel, B | 17001 | 1988 | Costa Rica | MEXU |
| *Hiraea smilacina* Standl. | Manhães, VC | 516 | 2014 | Brazil | VIES |
| *Hiraea smilacina* Standl. | Pereira, OJ | 7663 | 2008 | Brazil | SAMES |
| *Hiraea smilacina* Standl. | Ribeiro, M | 267 | 2010 | Brazil | SAMES |
| *Hiraea smilacina* Standl. | Kollmann, L | 934 | 1998 | Brazil | MBML |
| *Lophanthera hammelii* W.R. Anderson | Hammel, B | 9397 | 1980 | Costa Rica | MICH |
| *Lophopterys floribunda* W.R.Anderson & C.C.Davis | Leoni, LS | 4739 | 2001 | Brazil | RB |
| *Lophopterys floribunda* W.R.Anderson & C.C.Davis | Acácio, G | 96 | 1993 | Brazil | VIES |
| *Lophopterys floribunda* W.R.Anderson & C.C.Davis | Broggio, IS | 242 | 2016 | Brazil | HUENF |
| *Lophopterys floribunda* W.R.Anderson & C.C.Davis | Manhães, VC | 516 | 2014 | Brazil | VIES |
| *Lophopterys floribunda* W.R.Anderson & C.C.Davis | Kollmann, L | 3267 | 2000 | Brazil | MBML |
| *Microsteira ambovombensis* Arènes | McPherson, G | 14360 | 1989 | Madagascar | K |
| *Mionandra camareoides* Griseb. | Bastián, E | 10 | 1985 | Bolivia | MICH |
| *Mionandra camareoides* Griseb | Pedersen, TM | 13955 | 1984 | Argentina | MICH |
| *Mionandra camareoides* Griseb | Anderson, WR | 13585 | 1990 | Argentina | MICH |
| *Mionandra camareoides* Griseb | Wood, JRI & Goyder, DJ | 16763 | 2001 | Bolivia | MICH |
| *Mionandra camareoides* Griseb | Anderson, WR | 12342 | 1982 | Argentina | MICH |
| *Philgamia glabrifolia* Arènes | Andriamiharivo, TH | 80 | 2003 | Madagascar | P |
| *Philgamia glabrifolia* Arènes | Dorr, LJ | 3867 | 1985 | Madagascar | US |

**Table S3.2.** Continued

| **Species** | **Collector** | **Collector number** | **Year** | **Country** | **Herbarium code** |
| --- | --- | --- | --- | --- | --- |
| *Philgamia glabrifolia* Arènes | Capuron, R | 11.559 | 1955 | Madagascar | MICH |
| *Philgamia glabrifolia* Arènes | Lowry, PP II & Schatz, GE | 4825 | 1997 | Madagascar | MICH |
| *Philgamia glabrifolia* Arènes | Zhang, WH | 129 | 2008 | Madagascar | MICH |
| *Psychopterys dipholiphylla* (Small) W.R. Anderson & S. Corso | Sánchez, L; Velasco, G &Trujillo, V | 580 | 2014 | Mexico | US |
| *Psychopterys dipholiphylla* (Small) W.R. Anderson & S. Corso | Lyonnet, P | 3099 | 1940 | Mexico | US |
| *Psychopterys dipholiphylla* (Small) W.R. Anderson & S. Corso | Hinton, GB | 7498 | 1935 | Mexico | US |
| *Psychopterys dipholiphylla* (Small) W.R. Anderson & S. Corso | Hinton, GB | 7513 | 1935 | Mexico | US |
| *Psychopterys dipholiphylla* (Small) W.R. Anderson & S. Corso | Rzedowski, J | 27067 | 1970 | Mexico | US |
| *Ryssopterys intermedia* Hochr. | Hochreutiner, BPG | 35 | 1904 | Indonesia | P |
| *Tetrapterys discolor* (G.Mey.) DC. | Cordeiro, MR | 478 | 1975 | Brazil | IAN |
| *Tetrapterys discolor* (G.Mey.) DC. | McDowell, T | 2202 | 1990 | Guyana | MICH |
| *Tetrapterys discolor* (G.Mey.) DC. | Oliveira, E | 3365 | 1965 | Brazil | IAN |
| *Tetrapterys discolor* (G.Mey.) DC. | Silverstone-Sopkin, FA | 3521 | 1988 | Colombia | MICH |
| *Tetrapterys discolor* (G.Mey.) DC. | Plowman, T | 5815 | 1976 | Peru | MICH |
| *Tetrapterys phlomoides* (Spreng.) Nied. | Usteri, A | 12015 | 1906 | Brazil | NY |
| *Tetrapterys phlomoides* (Spreng.) Nied. | de Barros, AAM | 5409 | 2017 | Brazil | RB |
| *Tetrapterys phlomoides* (Spreng.) Nied. | Santos, MG | 460 | 1995 | Brazil | RB |
| *Tetrapterys phlomoides* (Spreng.) Nied. | Hatschbach, G | 75073 | 2003 | Brazil | MICH |
| *Tetrapterys phlomoides* (Spreng.) Nied. | Dias, MC | 2669 | 1986 | Brazil | MICH |
| *Triopterys paniculata* Small | Abbott, WL | 1064 | 1921 | Dominican Republic | US |
| *Triopterys paniculata* Small | Liogier, AH | 15283 | 1969 | Dominican Republic | US |
| *Triopterys paniculata* Small | Proctor, GR | 1103 | 1955 | Haiti | US |
| *Triopterys paniculata* Small | Greuter, WR; | 27104 | 2010 | Cuba | US |
| *Triopterys paniculata* Small | Liogier, AH | 14237 | 1969 | Dominican Republic | US |

**Reviewed literature:**

Almeida RF. Taxonomic revision of *Amorimia* W.R. Anderson (Malpighiaceae). Hoehnea. 2018; 45(2):238-306.

Almeida RF, Guesdon IR, Pace MR, Meira RMS. Taxonomic revision of *Mcvaughia* W.R. Anderson (Malpighiaceae): notes on vegetative and reproductive anatomy and the description of a new species. PhytoKeys. 2019; 117: 45–72.

Anderson C. A monograph of the genus *Peixotoa* (Malpighiaceae). Contr. Univ. Michigan Herb. 1982; 15:1-92.

Anderson C. Revision of *Thryallis* (Malpighiaceae). Contr. Univ. Michigan Herb. 1995; 20:3-14.

Anderson C. Monograph of *Stigmaphyllon* (Malpighiaceae). Syst. Bot. Monogr. 1997; 51:1-313.

Anderson C. Revision of *Galphimia* (Malpighiaceae). Contr. Univ. Michigan Herb. 2007; 25:1-82.

Anderson C, Anderson WR. Revision of *Mezia* (Malpighiaceae). Edinburgh J. Bot. 2018; 75: 321-376.

Anderson C, Anderson WR, Davis CC: Malpighiaceae. 2006-. https://webapps.lsa.umich.edu/herbarium/malpigh/. Accessed 12 May 2021.

Anderson WR. The taxonomy of *Acmantherea* (Malpighiaceae). Contr. Univ. Mich. Herb. 1975; 11(2):41-50.

Anderson WR. Malpighiaceae. In Flora of the Guyana Highland. Mem. New York Bot. Gard. 1981; 32:21-305.

Anderson WR*. Lophanthera*, a genus of Malpighiaceae new to Central America. Brittonia. 1983; 35:37-41.

Anderson WR. Notes on neotropical Malpighiaceae-II. Contr. Univ. Mich. Herb. 1987; 16:55-108.

Anderson WR. Malpighiaceae. In Howard RA, editor. Flora of the Lesser Antilles. 1988; 4:596-633.

Anderson WR. Notes on neotropical Malpighiaceae-III. Contr. Univ. Mich. Herb. 1990; 17:39-54.

Anderson WR. Notes on neotropical Malpighiaceae-VI. Contr. Univ. Michigan Herb. 1997; 21:37-84.

Anderson WR. Malpighiaceae. In Zuloaga FO, Morrone O, editors. Catálogo de las Plantas Vasculares de la República Argentina II, Fabaceae-Zygophyllaceae (Dicotyledoneae). Monogr. Syst. Bot. Missouri Bot. Gard. 1999a; 74: 804-813.

Anderson WR. Malpighiaceae. In Jorgensen PM, León-Yáñez S, editors. Catalogue of the Vascular plants of Ecuador. Monogr. Syst Bot. Missouri Bot. Gard. 1999b; 75:544-548.

Anderson WR. Malpighiaceae. In Berry PE, Yatskievych K, Holst BK, editors. Flora of the Venezuelan Guyana. Vol 6. 2001a; 82-185.

Anderson WR. Malpighiaceae. In Stevens WD, Ulloa C, Pool A, Montiel OM, editors. Flora de Nicaragua. Monogr. Syst. Bot. Missouri Bot. Gard. 2001b; 85(2):1256-1293.

Anderson WR. Eight segregastes from the Neotropical genus *Mascagnia* (Malpighiaceae). NOVON. 2006; 16:168-204.

Anderson WR. Malpighiaceae. In Hammel BE editor. Manual de plantas de Costa Rica. Vol 6. Monogr. Syst Bot. Missouri Bot. Gard. 2007; 111:253-312.

Anderson WR. Origins of Mexican Malpighiaceae. Act Bot Mex. 2013; 104:107-156

Anderson WR, Davis CC. Monograph of *Lophopterys* (Malpighiaceae) Contr. Univ. Michigan Herb. 2001; 23: 83-105.

Anderson WR, Davis CC. Generic adjustments in neotropical Malpighiaceae. Contr. Univ. Michigan Herb. 2007; 25:137-166.

Anderson WR, Gates B. *Barnebya,* a new genus of Malpighiaceae from Brazil. Brittonia. 1981; 33(3):275-284

Arènes J. Malpighiacées. In Humbert H, editor. Flore de Madagascar et des Comores-108. Muséum National d'Histoire Naturelle, Paris. 1950. 183p.

Cuatrecasas J. Prima Flora Colombiana. Malpighiaceae. Webbia. 1958; 13:343-664.

Cuatrecasas J. Croat TB. Malpighiaceae. In Woodson RE, Schery, RW, editors. Flora of Panama-Part VI. Ann. Missouri Bot. Gard. 1981; 67:851945.

Davis CC. *Madagasikaria* (Malpighiaceae): a new genus from Madagascar with implications for floral evolution in Malpighiaceae. Amer. J. Bot. 2002; 89:699706.

De Almeida RF, Pellegrini MO. Synopsis of *Bunchosia* Kunth (Malpighiaceae) from the Atlantic Forest. Phytotaxa. 2016; 257(2):158-166.

Brazil Flora G. Brazilian Flora 2020 project - Projeto Flora do Brasil 2020. Instituto de Pesquisas Jardim Botanico do Rio de Janeiro. 2020- <http://floradobrasil.jbrj.gov.br/>. Accessed on: 15 Aug. 2021.

Gates B. *Banisteriopsis, Diplopterys* (Malpighiaceae). Fl. Neotropica. 1982; 30:1-238.

González Gutiérrez PA. A revision of Cuban *Bunchosia* (Malpighiaceae), with description of a new subspecies from Hispaniola. Willdenowia. 2010; 40(1):51-56.

Guesdon IR, Amorim AM, Meira RMSA. Functional role and evolutionary contributions of floral gland morphoanatomy in the Paleotropical genus *Acridocarpus* (Malpighiaceae). PLoS One. 2019; 14(9), e0222561.

Jacobs M. Malpighiaceae. In Steenis CGGJ, Steenis-Kruseman MJ, editors. Flora Malesiana, ser. 1. 1955; 5(2):125145.

Johnson DM. Revision of the neotropical genus *Callaeum* (Malpighiaceae). Syst. Bot. 1986; 11:335-353.

Launert E. Malpighiaceae. In Flora of tropical East Africa. London: Crown Agents for Overseas Governments & Administrations. 1968; 24 pp.

León-Velasco ME. Malpighiaceae. In Jiménez J, Fonseca RM, Martínez M, editors. Flora de Guerrero. No. 61. CDMX: UNAM FC. 2014. 128 pp

Macbride JF. Malpighiaceae. In Flora of Peru, Field Mus. Nat. Hist., Bot. Ser. 1950; 13(3:3): 781-871.

Mamede MCH. Revisão do gênero *Camarea* Saint-Hilaire (Malpighiaceae). Hoehnea 1990; 17:1-134.

Vivaldi JL. New Taxa of *Malpighia* (Malpighiaceae) from Mexico and the West Indies. Brittonia. 1984; 63:330-332.
