# Supplementary material for: Phloem wedges in Malpighiaceae: origin, structure, diversification, and systematic relevance
Source: EvoDevo. 2022 Apr 28;13:11. doi: 10.1186/s13227-022-00196-3 (PMC9052467; doi:10.1186/s13227-022-00196-3)
Supplement: Supplementary file 4 — Additional file 4: Table S4.1 Hypothesis test comparing the log-likelihood and Akaike score (AIC) between equal rates (ER), and all rates different (ARD) transition models. Table S4.2 Stochastic character map results. Changes between each character state along the Malpighiaceae phylogeny. Table S4.3 Pagel’s 1994 Test of Correlated Evolution Results. Table S4.4 Results from Pagel’s λ and Blomberg’s K tests for phylogenetic signal. [file 13227_2022_196_MOESM4_ESM.docx]

**Additional file 4**

**Article title**: Phloem wedges in Malpighiaceae: origin, structure, diversification, and systematic relevance

**Authors:** Quintanar-Castillo A and Pace MR, 2022

**Model selection**

**Table S4.1.** Comparison between fit models that can explain the history and current distribution of the assessed characters (habit and phloem wedges presence). Hypothesis test comparing the log-likelihood and Akaike score (AIC) between equal rates (ER), and all rates different (ARD) transition models before stochastic character mapping.

| **Trait** | **Log-likelihoods** | **AIC** | **p-value: ARD vs ER substitution models** | **Best Fit Model** | **Number of states** |
| --- | --- | --- | --- | --- | --- |
| **Habit** |  |  | 0.00114 | ARD | 2 |
| ER | -70.7739 | 143.5479 |  |  |  |
| ARD | -65.4841 | 134.9683 |  |  |  |
| **Phloem wedges in stem** |  |  | 0.00644 | ARD | 2 |
| ER | -70.3141 | 142.6282 |  |  |  |
| ARD | -66.6032 | 137.2065 |  |  |  |


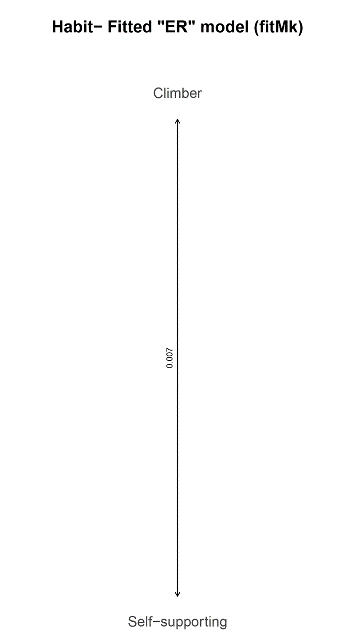

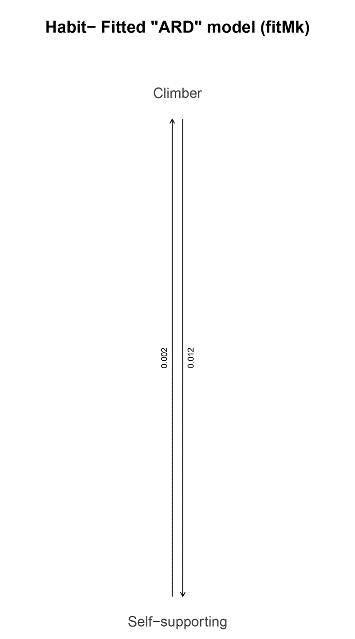

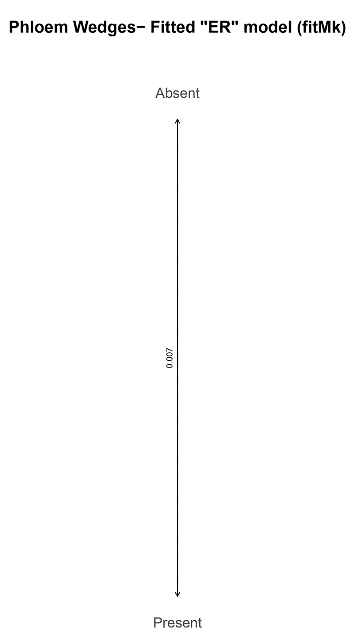

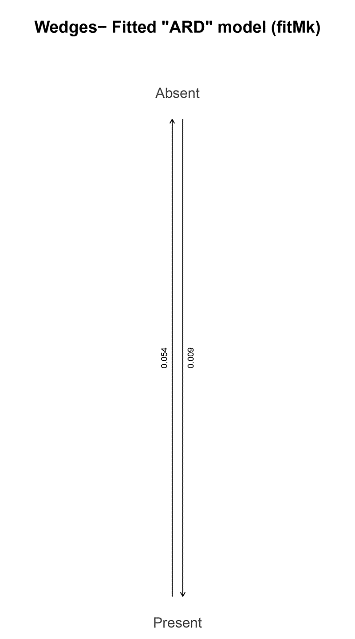


**Stochastic mapping with 100 simulations**

**Table S4.2** - Changes between each character state along the Malpighiaceae phylogeny obtained from stochastic character mapping with 100 iterations and performed on MCC tree.

| **Changes between character states** | **Number of changes in MCC with 100 iterations** |
| --- | --- |
| **Habit** |  |
| Self-supporting 🡪 Lianescent | 2.88 |
| Lianescent 🡪 Self-supporting | 17.2 |
| Total | 20.08 |
| **Phloem wedges in stem** |  |
| Phloem wedges present 🡪 Phloem wedges absent | 26.6 |
| Phloem wedges absent 🡪 Phloem wedges present | 22.18 |
| Total | 48.78 |


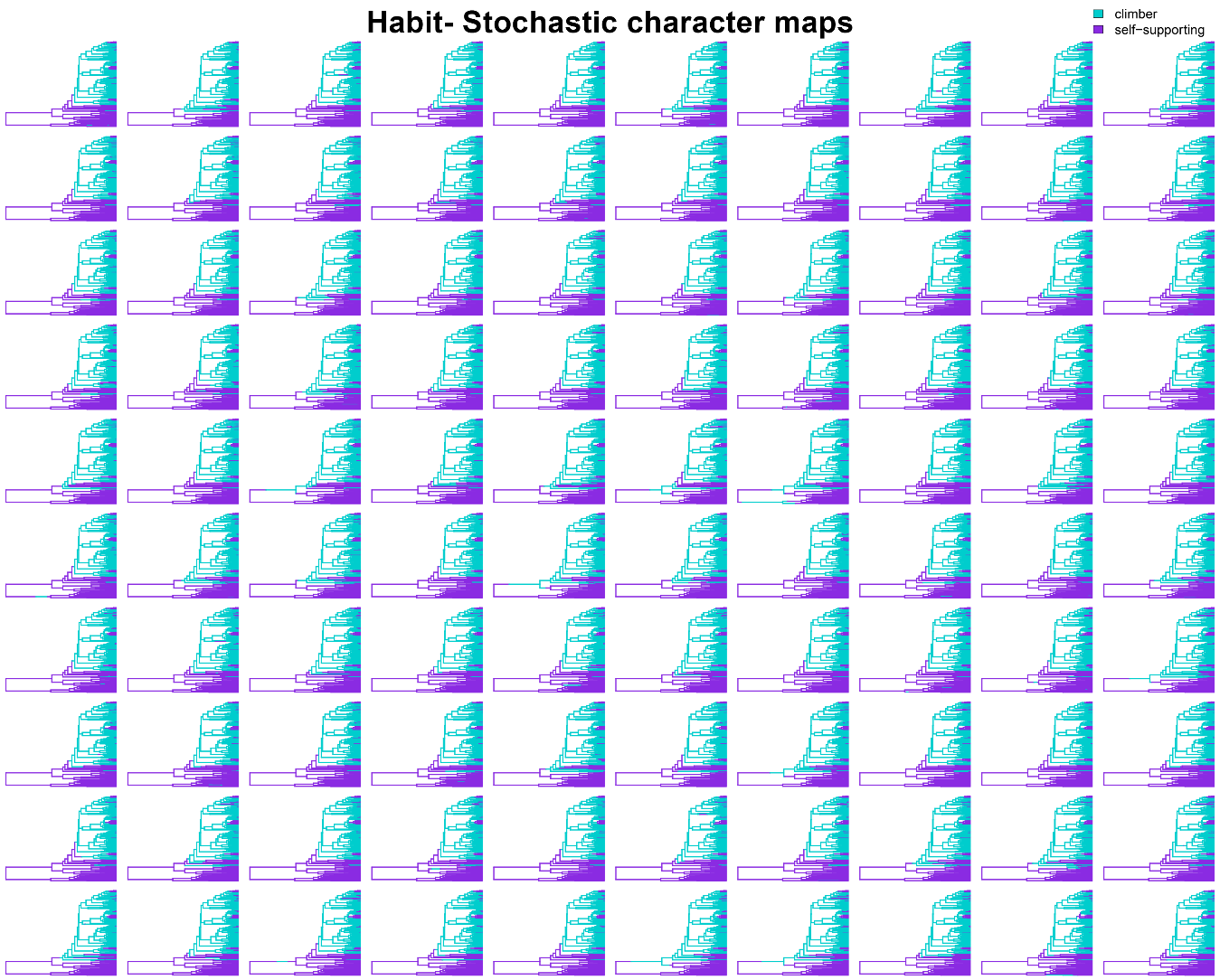


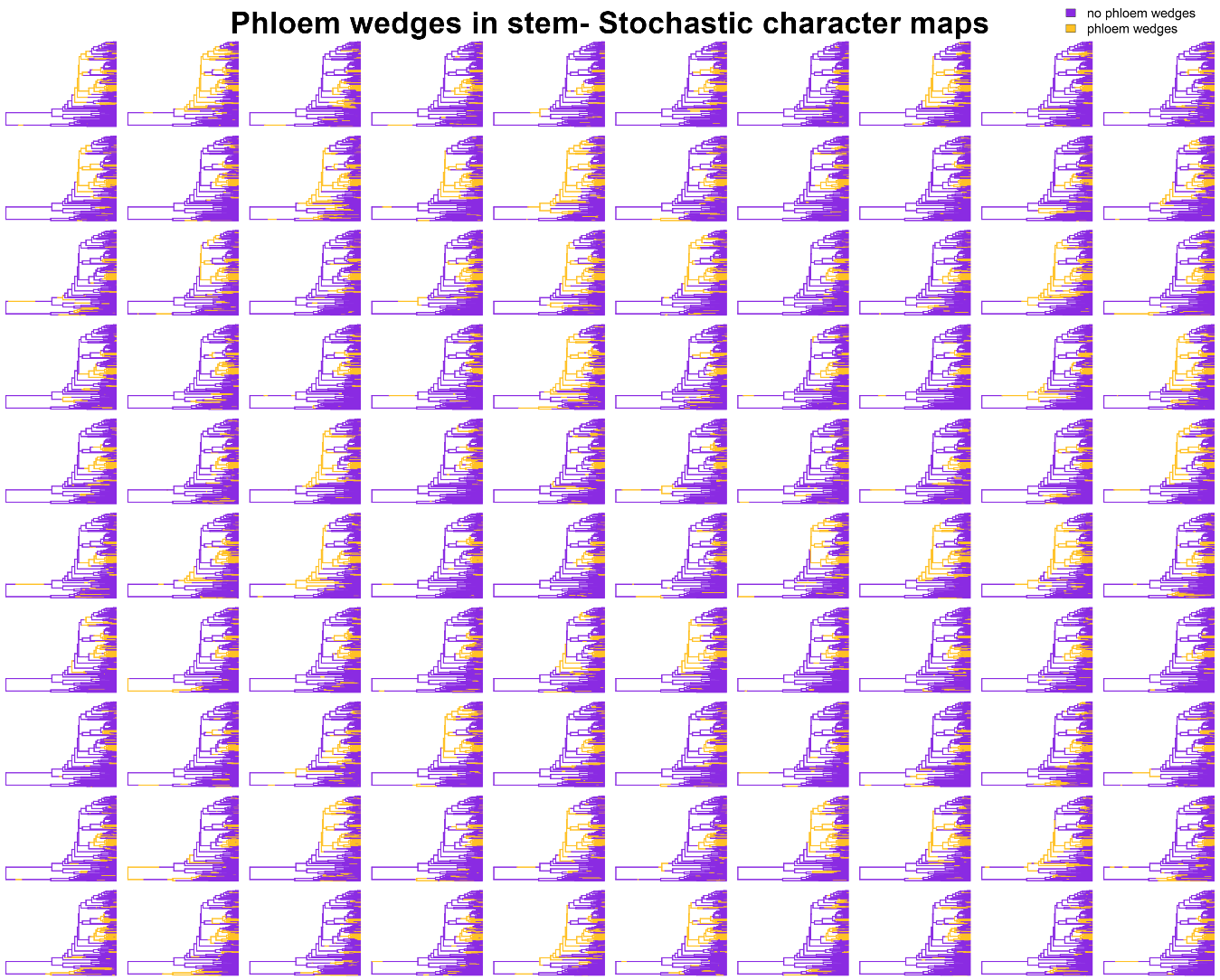


**Pagel’s 1994 Test of Correlated Evolution Results**

**Table S4.3.** Results of Pagel’94 correlation test [1] between habit and phloem wedges in stem, using all rates different (ARD) transition model.

| **Trait 1** | **Trait 2** | **Substitution model** | **Model fit** | | | | **Hipotesis test results** | |
| --- | --- | --- | --- | --- | --- | --- | --- | --- |
|  |  |  | *Independent* | | *Dependent* | | *Likelihood-ratio* | *p-value* |
|  |  |  | *Log-likelihood* | *AIC* | *Log-likelihood* | *AIC* |  |  |
| Habit (climber, self-supporting) | Phloem wedges in stems  (present, absent) | ARD | -132.2663 | 272.5325 | -117.7332 | 251.4665 | 29.0661 | 7.58E-06 |


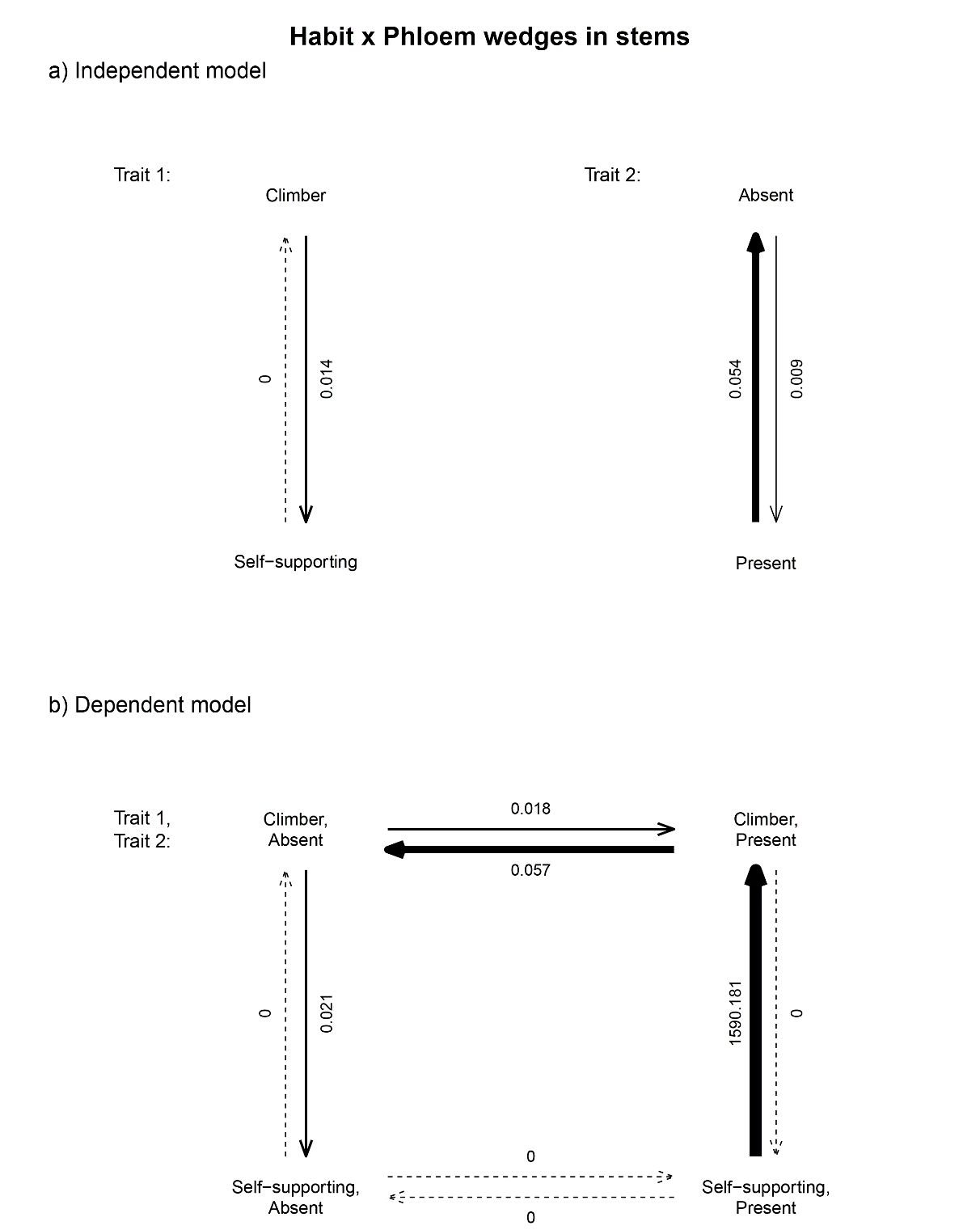


Pagel's (1994) analysis for detecting correlated evolution of habit and phloem wedges in the stem. Arrows represent the direction of transition and values on arrows indicate transition rates.

**Phylogenetical signal**

**Table S4.4.** Results from Pagel’s λ [2] and Blomberg’s *K* [3] tests for phylogenetic signal of each character.

| **Character** | **Pagel’s λ** | | | **Blomberg’s K** | |
| --- | --- | --- | --- | --- | --- |
|  | *lamba* | *p-value* | *likelihood ratio test* | *K* | *p-value (based on 1000 randomizations)* |
| Habit | 0.8646 | 2.63E-24 | -66.5389 | 0.6121 | 0.001 |
| Phloem wedges in stem | 0.8078 | 4.026E-09 | -65.8802 | 0.2189 | 0.001 |

The characters tested in this study showed strong phylogenetic signals based on Pagel’s λ test, however, the result of Blomberg’s K test of each trait showed a weak phylogenetic signal, in particular the phloem wedges in the stem. Pagel's λ was chosen over Blomberg’s K index since the former generally performs better than the latter in detecting phylogenetic signal, under a Brownian-motion evolution (BM) [4]


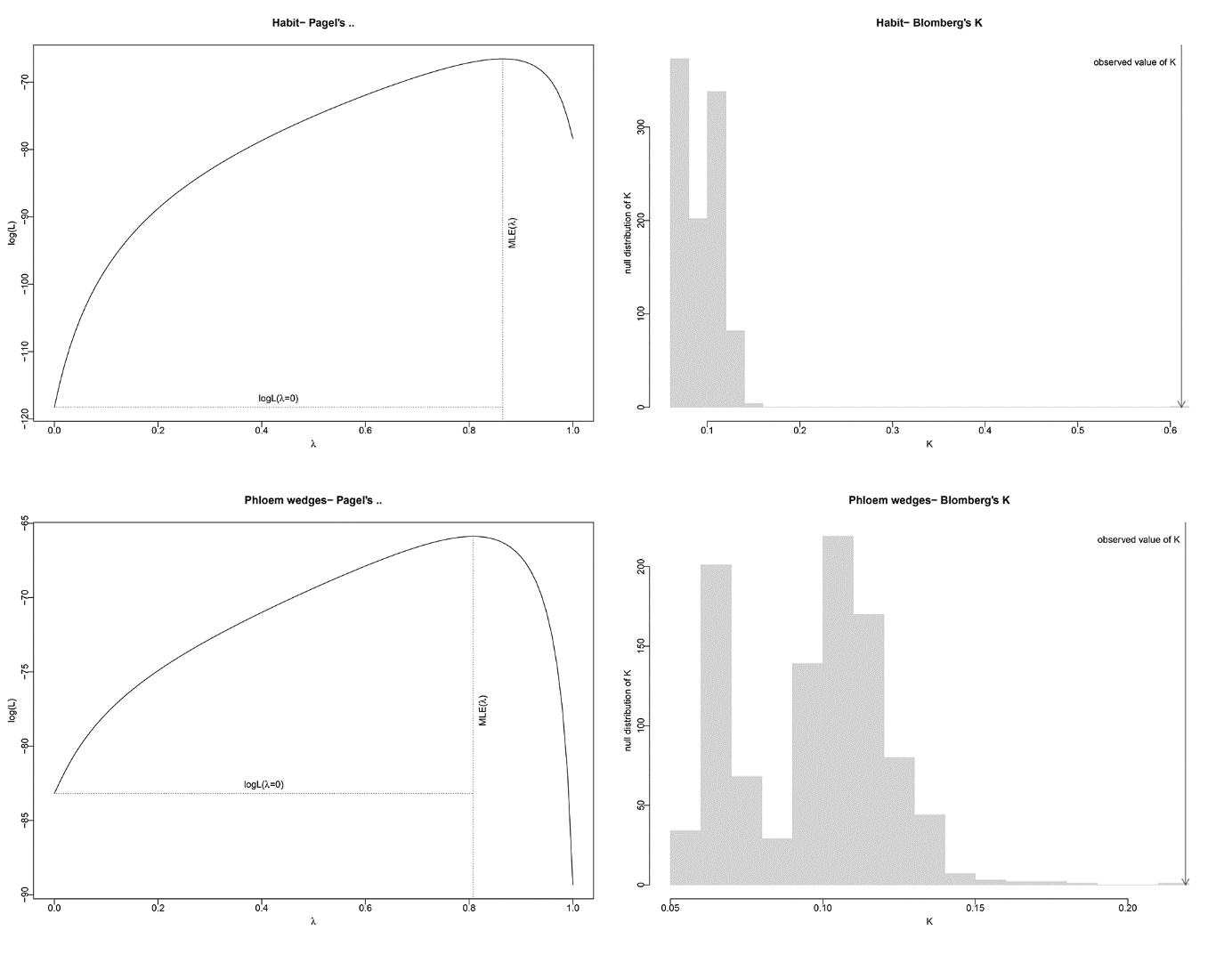


**References**

1. Pagel M. Detecting correlated evolution on phylogenies: a general method for the comparative analysis of discrete characters. Proc. R. Soc. London. Ser. B Biol. Sci. 1994; 255: 37-45.
2. Pagel M. Inferring the historical patterns of biological evolution, Nature. 1999; 401: 877-884.
3. Blomberg SP, Garland T, Ives AR. Testing for phylogenetic signal in comparative data: behavioral traits are more labile. Evolution. 2003; 57: 717-745.
4. Münkemüller T, Lavergne S, Bzeznik B, Dray S, Jombart T, Schiffers K, Thuiller W. How to measure and test phylogenetic signal. Methods Ecol. Evol. 2012; 3: 743-756.
